# A dicoordinate gold(i)–ethylene complex†

**DOI:** 10.1039/d1cc02769g

**Published:** 2021-08-11

**Authors:** Miquel Navarro, Juan Miranda-Pizarro, Juan J. Moreno, Carlos Navarro-Gilabert, Israel Fernández, Jesús Campos

**Affiliations:** Instituto de Investigaciones Químicas (IIQ), Departamento de Química Inorgánica and Centro de Innovación en Química Avanzada (ORFEO-CINQA), Consejo Superior de Investigaciones Científicas (CSIC) and University of Sevilla Sevilla 41092 Spain jesus.campos@iiq.csic.es; Departamento de Química Orgánica I and Centro de Innovación en Química Avanzada (ORFEO-CINQA), Facultad de Ciencias Químicas, Universidad Complutense de Madrid Madrid 28040 Spain israel@quim.ucm.es

## Abstract

The use of the exceptionally bulky tris-2-(4,4′-di-*tert*-butylbiphenylyl)phosphine ligand allows the isolation and complete characterization of the first dicoordinate gold(i)–ethylene adduct, filling a missing fundamental piece on the organometallic chemistry of gold. Besides, the bonding situation of this species has been investigated by means of state-of-the-art Density Functional Theory (DFT) calculations indicating that π-backdonation plays a minor role compared with tricoordinate analogues.

## Abstract 2

The use of the exceptionally bulky tris-2-(4,4′-di-*tert*-butylbiphenylyl)phosphine ligand allows the isolation and complete characterization of the first dicoordinate gold(i)–ethylene adduct, filling a missing fundamental piece on the organometallic chemistry of gold.

## Graphical abstract

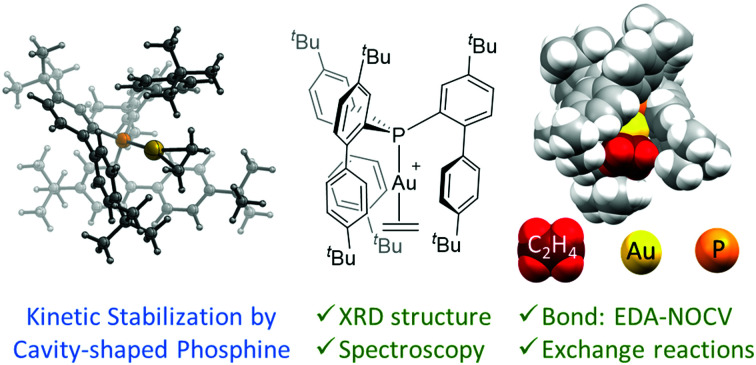

Homogenous gold catalysis has become one of the most powerful tools in organic synthesis due to the prowess of Au(i) towards the electrophilic activation of unsaturated hydrocarbons.^1^ π-Complexes of gold are generally proposed as key intermediates in the functionalisation of alkenes, alkynes, dienes or allenes in a wide range of catalytic processes, including hydrogenation, hydroamination, oxidation, diarylation, heteroarylation or cycloadditions.^2^ Thus, the isolation of gold π-complexes holds an intrinsic interest associated with their catalytic relevance and has provided valuable insight during recent years.^3^

Cationic, dicoordinate gold(i) π-complexes of substituted alkenes, stabilized by monodentate N-heterocyclic carbenes (NHCs)^4^ or phosphines,^5^ have been isolated and characterized. However, despite significant synthetic efforts, dicoordinate ethylene complexes remain unknown.^6^ Only by using N- and P-based bidentate ligands the related tricoordinate gold(i) π-ethylene adducts (Fig. 1)^7^ have been detected and characterized. Nonetheless, despite the growing interest for bidentate ligands in gold(i) chemistry,^8^ these species do not represent key intermediates in gold catalysis. The increased backdonation from Au to the ethylene π*(C=C) orbital in tricoordinate species is prevented in the so far elusive dicoordinate gold(i)–ethylene adducts, which have remained an unsolved synthetic challenge.

**Fig. 1:**
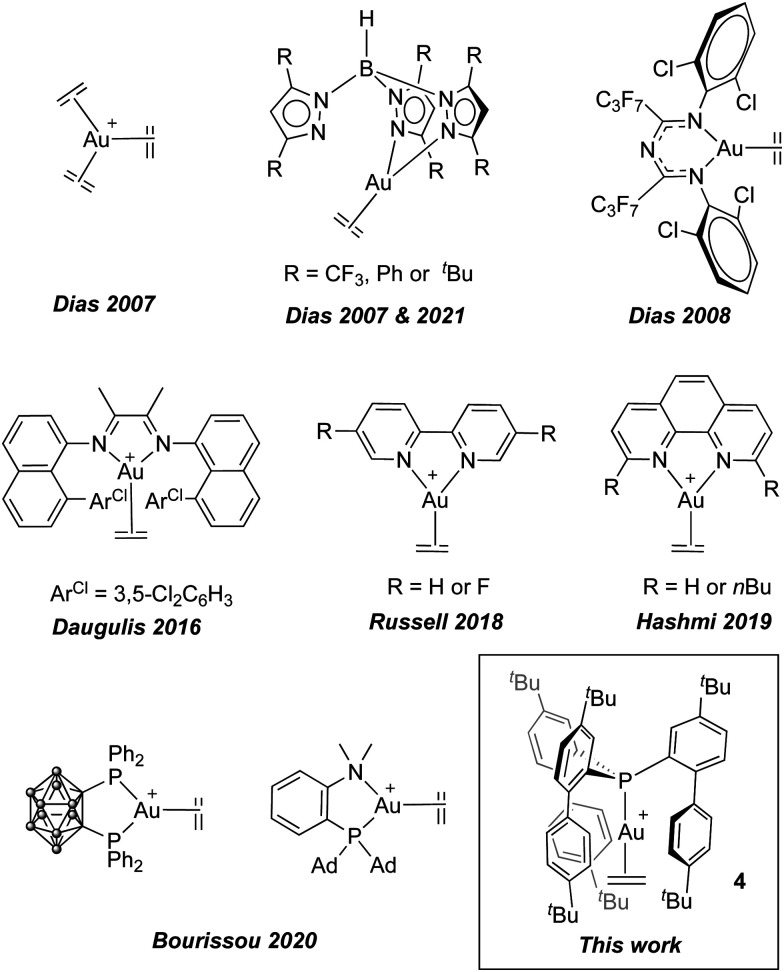
Structurally authenticated gold(i) ethylene complexes, all based on tricoordinate gold, and the first dicoordinate gold(i) ethylene complex reported herein.

In addition to the synthesis of highly active gold catalysts,^9^ the use of sterically shielding ligands has become a successful strategy to stabilize exotic gold(i) species.^10^ In this regard, our group has recently capitalized on the use of sterically demanding phosphine ligands to access unusual Au(i) structures, including the isolation of bridged cationic digold complexes^11^ and the reactivity of gold species as FLP constituents^12^ and in C–C bond formation processes.^13^ In this study, we have employed the ligand tris-2-(4,4′-di-*tert*-butylbiphenylyl)phosphine (**1**),^14^ previously reported by the group of Straub, which allowed us to isolate and structurally characterize the first dicoordinate gold(i)–ethylene complex. This species can be regarded as the simplest model reported to date for the isolobal ethenium cation (C_2_H_5_^+^).^15^

Treatment of [AuCl(THT)] (THT = tetrahydrothiophene) with phosphine **1**^10*c*^ forms the air-stable, neutral phosphine chloride complex **2** (96% yield). Single-crystal X-ray diffraction analysis (Fig. S27, ESI†) confirmed that the *ortho*-aryl groups of the phosphine ligand are directed towards the gold atom, ensuing great steric protection around it. The percent buried volume (%*V*_Bur_)^16^ of the phosphine yielded a large parameter of 67.0 (Fig. S28, ESI†), which is notably higher than other bulky NHC^17^ and phosphine^18^ ligands.

To access an electrophilic [P–Au]^+^ fragment amenable to ethylene coordination, complex **2** was reacted with AgSbF_6_ as a halogen abstractor in dichloromethane. Although chloride capture was not achieved, a new species was detected by ^31^P{^1^H} NMR spectroscopy at 4.6 ppm (**3** in Scheme 1; *cf.*^31^P{^1^H} *δ* 9.5 ppm for **2**). X-Ray diffraction analysis revealed the formation of the unusual trimetallic complex **3** bearing two bridging Ag centres between the Au and Cl atoms (Fig. 2). The flexibility of the cavity around gold allows for allocating the two silver atoms which are further stabilized by two π-interactions for each metal site with the *ortho*-aryl rings (*d*_AgC_ ≈ 2.5–2.7 Å). Related bimetallic species are assumed to be intermediates during metathesis reactions with silver salts, though we could not detect any hint of AgCl precipitation.^19^ Alternatively, when the reaction was performed under ethylene atmosphere at −30 °C, instantaneous precipitation of AgCl was observed. Monitoring the reaction by ^31^P{^1^H} NMR spectroscopy revealed quantitative formation of a new gold(i) species resonating at 13.1 ppm (Scheme 1). Filtering the reaction mixture through a short pad of Celite afforded the pure gold(i)–ethylene complex **4**, which is surprisingly stable in solution at room temperature under inert atmosphere and showed only slow decomposition during solvent evaporation and in the solid-state. Its ^1^H and ^13^C{^1^H} NMR spectra display a new set of well-defined signals indicative of ethylene coordination to gold, including a distinctive AA′BB′ pattern in the ^1^H NMR spectrum at 3.79 and 3.66 ppm (Fig. S11, ESI†).

**Scheme 1:**
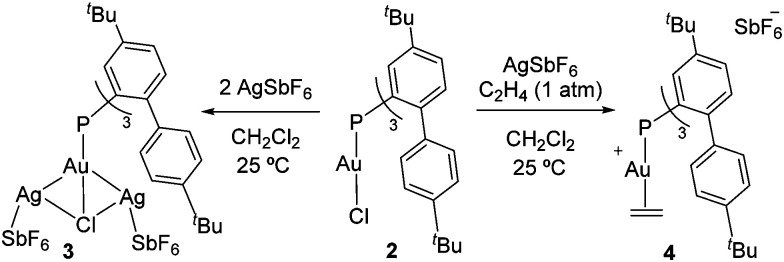
Synthesis of trimetallic gold–disilver complex **3** and gold(i)–ethylene adduct **4**.

**Fig. 2:**
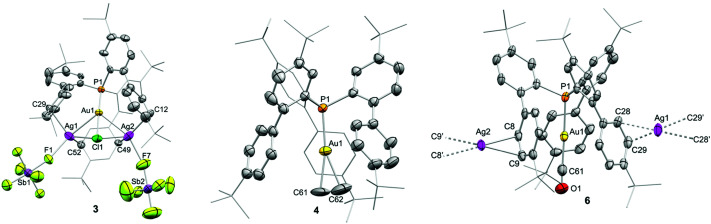
ORTEPs of **3** (one of two independent molecules), **4** and **6** (polymeric structure due to co-crystallisation with silver; C8′/C9′ (−*x*, 1 − *y*, −*z*) and C28/C29′ (1 − *x*, 1 − *y*, 1 − *z*) are at equivalent positions). Counteranion, solvent molecules and hydrogen atoms are excluded for clarity, while *tert*-butyl groups and one biaryl fragment are represented in wireframe format. Thermal ellipsoids are set at 50% probability. Selected bond lengths (Å) and angles (°): compound **3**, P1–Au1, 2.246(4); Au1–Ag1, 2.8212(15); Au1–Ag2, 2.8015(16); Au1–Cl1, 2.316(4); Ag1–Cl1, 2.735(5); Ag2–Cl1, 2.712(5); Ag1–C29, 2.549(15); Ag2–C12, 2.584(18); Ag1–C52, 2.709(16); Ag2–C49, 2.600(18); Ag01–Au1–Ag2, 103.78(6); Cl1–Au1–Ag1, 63.43(12); C29–Ag1–Au1, 94.0(4); C52–Ag1–Au1, 83.7(3); compound **4** (two independent molecules per asymmetric unit), P1–Au1, 2.2811(8), 2.2830(11); Au1–C61, 2.197(8), 2.2197(7); Au1–C62, 2.218(8), 2.214(8); P1–Au1–C61, 165.3(4), 169.8(3); P1–Au1–C62, 165.7(3), 169.8(3). Compound **6**, P1–Au1, 2.2952(15); Au1–C61, 1.954(8); C61–O1, 1.075(9); P1–Au1–C61, 176.6(2).

The aforementioned ^1^H NMR signals are considerably shifted from those in free ethylene (5.43 ppm), which contrasts with the minimal variation of their corresponding ^13^C{^1^H} NMR resonances (*δ* = 110.2 (^2^*J*_CP_ = 9 Hz); *cf.* ethylene: *δ* = 116.8 ppm). A comparable upfield ^1^H NMR shift has been observed in tricoordinate gold(i) ethylene adducts, which induce some metallacyclic character on the coordinated ethylene as a result of significant Au → ethylene π-backdonation.^6^ However, considering the limited Au → ethylene π-backdonation in **4** (*vide infra*), we attribute the marked upfield shift to the close proximity of the coordinated ethylene molecule to three *ortho*-aryl groups of the phosphine, becoming susceptible of a substantial aromatic ring current effect (see ESI†).

Two different types of single crystals suitable for X-ray diffraction analysis were obtained by slow evaporation of pentane into concentrated dichloromethane solutions of complex **4**. The two structures present the gold centre in a linear environment with an ethylene molecule coordinating gold in an η^2^ fashion (Fig. 2 and Fig. S29, ESI†). While one molecular structure corresponds to the expected gold(i)–ethylene adduct **4** (Fig. 2), the other presents a dimeric structure with a bridging {Ag_2_(C_2_H_4_)(μ-H_2_O)_2_}^2+^ motif (**4′**); Fig. S29, ESI†) due to silver traces that were not completely removed during the reaction work-up. Since the ethylene molecule is somewhat disordered in **4**, we discuss its geometric parameters on the silver-containing structure **4′**, which is in perfect agreement with the computed geometry. The Au–C bond lengths (2.216(6) and 2.235(6) Å) are noticeably longer than those described for gold(i)–ethylene adducts bearing bidentate ligands (*cf.* Au–C lengths in Bourissou's systems in Fig. 1 span from 2.141 to 2.175 Å),^7^ but similar to cationic dicoordinate gold(i) π-complexes with other alkenes.^5^ The C=C double bond is slightly shorter than that of free ethylene (1.263(10) Å *vs.* 1.313 Å, respectively)^20^ and significantly shorter compared to the described tricoordinate gold(i) ethylene complexes (*cf.* 1.365(15) and 1.387(5) Å in Bourissou's compounds), indicative of poor Au → ethylene π-backdonation. In addition, both molecular structures showed that the *ortho*-aryl groups of the phosphine ligand are in relatively close proximity to the coordinated ethylene molecule, reinforcing the idea of a significant aromatic ring current effect affecting the ^1^H resonance frequency of the bound C_2_H_4_.

Through the years, the interest in the bonding between ethylene and cationic gold(i) has sparked several computational studies on the matter.^21^ These suggest that, although the interaction is mainly electrostatic, the orbital component can be rationalized in terms of the Dewar–Chatt–Duncanson model.^22^ To gain insight into the bonding situation of **4**, DFT calculations were carried out at the dispersion corrected ZORA-BP86-D3/TZVP//BP86-D3/def2-SVP level (see computational details in the ESI†). For comparison, we also analysed the bonding in the related gold(i)–ethylene complexes [(Ph_3_P)Au(C_2_H_4_)]^+^ (**A**) and [(NHC)Au(C_2_H_4_)]^+^ (**B**, NHC = 1,3-bis(2,6-dimethylphenyl)imidazol-2-ylidene) by means of the EDA-NOCV method. From the data in Table S3 (ESI†), it is confirmed that the main contribution to the bonding between the transition metal fragment and ethylene not only in **4** but also in **A** and **B** comes from the electrostatic attractions, which contribute *ca.* 56–59% to the total interaction. In all cases, two main orbital interactions dominate the total Δ*E*_orb_ term, namely the σ-donation from the doubly-occupied π(CH_2_=CH_2_) molecular orbital to the vacant σ*(Au–P/C) orbital (denoted as *ρ*_1_, Fig. 3) and the π-backdonation from an occupied d_π_(Au) atomic orbital to the π*(CH_2_=CH_2_) (denoted as *ρ*_2_). Our calculations indicate that the latter interaction is nearly half as strong as the former, thus supporting the above commented conclusion based on structural grounds on the weak Au → ethylene π-backdonation in **4**.

**Fig. 3:**
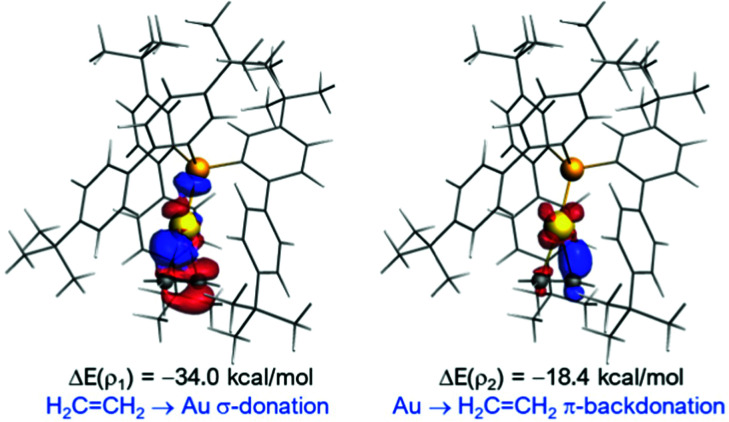
Contour plots of NOCV deformation densities Δ*ρ* and associated energies Δ*E*(*ρ*) in **4**. Electron-density charge flows in the direction red → blue.

The lability of the gold(i)–ethylene bond in complex **4** was also evaluated by several exchange experiments. First, 2D exchange spectroscopy (EXSY) experiments in CD_2_Cl_2_ evidenced chemical exchange between coordinated and free ethylene within the NMR time scale (Fig. S13, ESI†). An Eyring analysis in the temperature interval from −20 °C to 30 °C (Fig. S24, ESI†) provided the kinetic parameters Δ*H*^‡^ = 6.7 kcal mol^−1^ and Δ*S*^‡^ = −38.1 cal K^−1^ mol^−1^ corresponding to a 
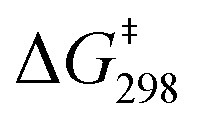
 for ethylene exchange of 18.0 kcal mol^−1^, which surpasses prior estimations based on bulkier olefins^5*a*^ and thus supports the notion of a remarkable kinetic stabilisation provided by the cavity-shaped phosphine.^23^ In fact, the large negative value of the entropic factor denotes an associative exchange mechanism, somewhat hindered by the shielding nature of the three *ortho*-aryl substituents. We decided to examine this process also by computational means and found a transition state for the associative process at 17.4 kcal mol^−1^ leading to a tricoordinate intermediate [P*Au(C_2_H_4_)_2_]^+^ at 12.7 kcal mol^−1^, in excellent agreement with our experimental studies. We further examined the lability of the gold–ethylene bond by reacting complex **4** with MeCN and CO to form the corresponding gold(i)–acetonitrile (**5**) and carbonyl (**6**) adducts, respectively. NMR exchange experiments revealed an affinity trend of MeCN > CO > ethylene (Scheme S1 and Fig. S25, ESI†).

Compounds **5** and **6** were fully characterized by NMR spectroscopy and X-ray diffraction techniques (Fig. 2 and ESI† for more details). The IR spectrum of **6** displays a strong absorption band at 2169 cm^−1^ which corresponds to the CO stretch. This value is relatively higher than free CO (*υ*_CO_ = 2143 cm^−1^) but slightly lower to related cationic NHC (2197 cm^−1^)^24*b*^ and phosphine (*υ*_CO_ = 2185 cm^−1^)^24*a*^ Au(i)–CO complexes. The bonding situation of **6** strongly resembles that of **4**, *i.e.* the electrostatic term dominates the total interaction between the gold(i)-fragment and CO (Table S3, ESI†). At variance, the strength of the Au → π*(CO) π-backdonation is nearly identical to the OC → Au σ-donation, which is not surprising due to the much higher π-acceptor nature of the CO ligand as compared to CH_2_=CH_2_.

In summary, we have synthesized and structurally characterized the first dicoordinate gold(i)–ethylene complex, thus filling a long-sought gap in the organometallic chemistry of Au(i). Exchange experiments demonstrate that the cavity provided by the extremely bulky phosphine ligand kinetically stabilizes the coordination of ethylene. Analysis of the bonding interactions by means of the EDA-NOCV method supports a major electrostatic (*i.e.* ionic) component and a minor role for π-backdonation in a linear coordination environment, which is markedly different to the situation found in related tricoordinate gold(i)–ethylene adducts.^7^ These results provide valuable insights for the underdeveloped functionalisation of ethylene mediated by gold.^25^

This work was supported by the European Research Council (ERC Starting Grant, CoopCat, Project 756575) and the Spanish Ministry of Science and Innovation (Projects PID2019-110856GA-I00, PID2019-106184GB-I00, RED2018-102387-T and FJC2018-035514-I (M. N.)). M. N. and J. J. M. acknowledge Junta de Andalucía for postdoctoral program (ref. DOC_00149 and DOC_00153).

## Conflicts of interest

There are no conflicts to declare.

## Supplementary Material

CC-057-D1CC02769G-s001

CC-057-D1CC02769G-s002
