# Supplementary material for: A dicoordinate gold(i)–ethylene complex
Source: Chem Commun (Camb). 2021 Aug 11;57(73):9280–3. doi: 10.1039/d1cc02769g (PMC8438763; doi:10.1039/d1cc02769g)
Supplement: CC-057-D1CC02769G-s001 [file CC-057-D1CC02769G-s001.pdf]

Electronic Supplementary Material (ESI) for Chemical Communications.
This journal is © The Royal Society of Chemistry 2021

Supporting Information

A Dicoordinate Gold(I)–Ethylene Complex

Miquel Navarro+,[a] Juan Miranda-Pizarro+,[a] Juan J. Moreno,[a] Carlos Navarro-Gilabert,[a] Israel Fernández*[b] and Jesús Campos*[a]

[a] Dr. Miquel Navarro, Juan Miranda-Pizarro, Dr. Juan J. Moreno, Carlos Navarro-Gilabert, Dr. Jesús Campos
Instituto de Investigaciones Químicas (IIQ), Departamento de Química Inorgánica and Centro de Innovación en Química Avanzada (ORFEO−CINQA)
Consejo Superior de Investigaciones Científicas (CSIC) and University of Sevilla, 41092 Sevilla, Spain
E-mail: jesus.campos@iiq.csic.es

[b] Prof. Dr. Israel Fernández
Departamento de Química Orgánica I and Centro de Innovación en Química Avanzada (ORFEO-CINQA)
Facultad de Ciencias Químicas, Universidad Complutense de Madrid, 28040-Madrid (Spain)
E-mail: israel@quim.ucm.es

[+] These authors contributed equally to this work.

1. Synthetic procedures **S2**
2. NMR spectroscopic experiments **S6**
3. IR spectrum of complex 6 .... **S20**
4. Crystal structure determinations **S22**
5. Buried volume analysis **S28**
6. Computational details… **S29**
7. References **S63**

1. Synthetic procedures

General considerations. Unless otherwise stated, all reactions and manipulations were carried out under an atmosphere of dry argon or nitrogen using standard Schlenk techniques or in a nitrogen glovebox. Solvents were distilled under inert atmosphere prior to use. Solution ^{1}H, ^{13}C and ^{31}P NMR spectra were recorded on Bruker AMX-300, DRX-400 or DRX-500 spectrometers at 298 K unless otherwise stated. Chemical shifts (δ) are expressed with a positive sign, in parts per million. ^{1}H and ^{13}C chemical shifts reported are referenced internally to residual protio (^{1}H) or deutero (^{13}C) solvent, while ^{31}P chemical shifts are relative to 85% H_3PO_4. The following abbreviations and their combinations are used: br, broad; s, singlet; d, doublet; t, triplet; m, multiplet. The ^{1}H and ^{13}C resonance signals were attributed by means of 2D HSQC and HMBC experiments. Infrared spectra were recorded on a Bruker Vector 22 spectrometer, and sample preparation was carried out in dry dichloromethane solution. For elemental analyses a LECO TruSpec CHN elementary analyser was utilized. Tris-2-(4,4'-di-tert-butylbiphenylyl)phosphine (**1**)[1] and [AuCl(tht)][2] (tht = tetrahydrothiophene) were synthesized as reported in the literature. All other reagents were used as received from commercial suppliers.

Compound 2

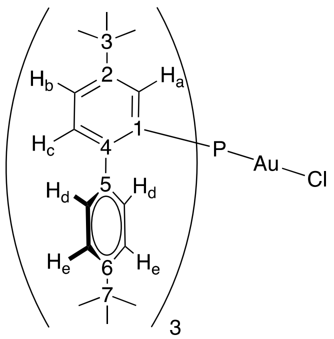

A solution of phosphine **1** (205 mg, 0.25 mmol) and [AuCl(tht)] (80 mg, 0.25 mmol) in CH_2Cl_2 (5 mL) was stirred at 0 ºC for 1 h. The solvent was then removed under vacuum and the crude solid was purified through column chromatography (SiO_2; Pentane/CH_2Cl_2 4:1), yielding complex **2** as a white solid (254 mg, 96%). Crystals suitable for X-ray diffraction were grown by slow evaporation of pentane into a dichloromethane solution of complex **2**. **Anal. Calcd.** for $C_{60}H_{75}AuClP$: C, 68.0; H, 7.13. **Found:** C, 68.0; H, 7.2. **^{1}H NMR** (400 MHz, CD_2Cl_2, 25 ºC) δ: 7.58 (dd, 3H, $^{3}J_{HH}$ = 8.0 Hz, $^{4}J_{HH}$ = 2.1 Hz, H_b), 7.35–7.26 (m, 6H, H_a, H_c), 7.09 (d, 6H, $^{3}J_{HH}$ = 8.0 Hz, H_e), 6.62 (d, 6H, $^{3}J_{HH}$ = 8.0 Hz, H_d), 1.27 (s, 27H, CH_3 (^{t}Bu$_3$)), 1.22 (s, 27H, CH_3 (^{t}Bu$_7$)). **^{13}C{^{1}H} NMR** (100 MHz, CD_2Cl_2, 25 ºC) δ: 151.4 (C_6), 150.6 (d, $^{3}J_{CP}$ = 7 Hz, C_2), 145.5 (d, $^{2}J_{CP}$ = 16 Hz, C_4), 138.2 (d, $^{3}J_{CP}$ = 7 Hz, C_5), 133.2 (d, $^{3}J_{CP}$ = 5 Hz, CH_a or CH_c), 132.5 (d, $^{3}J_{CP}$ = 8 Hz, CH_a or CH_c), 130.7 (d, $^{1}J_{CP}$ = 60 Hz, C_1), 129.7 (CH_d), 128.1 (CH_b), 125.7 (CH_e), 35.4 (C_3), 35.1 (C_7), 31.9 (C(CH_3)$_3$ (^{t}Bu$_7$)), 31.7 (C(CH_3)$_3$ (^{t}Bu$_3$)). **^{31}P{^{1}H} NMR** (162 MHz, CD_2Cl_2, 25 ºC) δ: 9.5.

Compound 3

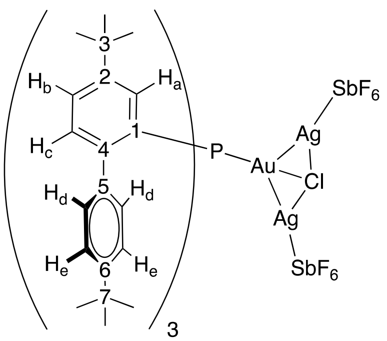

A solution of compound **2** (20 mg, 0.019 mmol), $AgSbF_6$ (14 mg, 0.040 mmol) in CH_2Cl_2 (1 mL) was stirred at room temperature for 1 h. The mixture was filtered through a short pad of Celite. The solvent was removed under vacuum, thus yielding complex **3** as a white solid in quantitative yield. Crystals suitable for X-ray diffraction were grown by slow diffusion of pentane into a concentrated dichloromethane solution of complex **3**. **^{1}H NMR** (400 MHz, CD_2Cl_2, 25 ºC) δ: 7.78 (dt, 3H, $^3J_{HH}$ = 8.1 Hz, $^4J_{HH}$ = $^5J_{HP}$ = 1.8 Hz, H_b), 7.51 (dd, 3H, $^3J_{HP}$ = 12.4 Hz, $^4J_{HH}$ = 1.8 Hz, H_a), 7.47 (dd, 3H, $^3J_{HH}$ = 8.1 Hz, $^4J_{HP}$ = 5.9 Hz, H_c), 7.38 (br d, 6H, $^3J_{HH}$ = 7.0 Hz, H_e), 6.85 (br, 6H, H_d), 1.34 (s, 27H, CH_3 (^{t}Bu$_7$)), 1.27 (s, 27H, CH_3 (^{t}Bu$_3$)). **^{13}C{^{1}H} NMR** (100 MHz, CD_2Cl_2, 25 ºC) δ: 154.4 (C_6), 152.8 (d, $^3J_{CP}$ = 8 Hz, C_2), 143.0 (d, $^2J_{CP}$ = 15 Hz, C_4), 140.3 (d, $^3J_{CP}$ = 7 Hz, C_5), 133.6 (d, $^3J_{CP}$ = 9 Hz, CH_a or CH_c), 133.4 (d, $^3J_{CP}$ = 5 Hz, CH_a or CH_c), 130.4 (CH_b), 129.9 (CH_d), 127.9 (d, $^1J_{CP}$ = 64 Hz, C_1), 124.9 (CH_d), 123.9 (CH_e), 35.9 (C_3), 35.7 (C_7), 32.3 (C(CH$_3$)$_3$ (^{t}Bu$_7$)), 31.6 (C(CH$_3$)$_3$ (^{t}Bu$_3$)). **^{31}P{^{1}H} NMR** (162 MHz, CD_2Cl_2, 25 ºC) δ: 4.6.

Compound 4

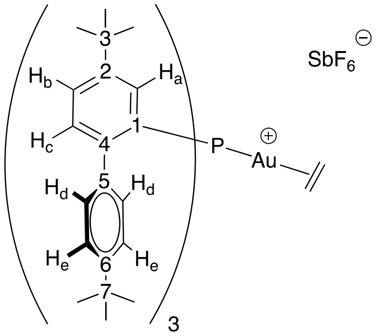

In a glovebox, a Schlenk flask was charged with silver hexafluoroantimonate (19 mg, 0.055 mmol) in dichloromethane (2 mL). Complex **2** (53 mg, 0.05 mmol) was transferred into a small glass vial and dissolved in dichloromethane (2 mL). The vial solution was loaded into a plastic syringe equipped with a stainless steel needle. Outside the glovebox, the Schlenk flask was cooled down to –30ºC. At this temperature the solution of complex **2** was added to the $AgSbF_6$ suspension while bubbling ethylene. The mixture was allowed to slowly warm up to room temperature, filtered through a short pad of Celite to remove the silver salts, and the solvent was removed under vacuum affording complex **4** (53 mg, 83%). Crystals suitable for X-ray diffraction were grown by slow diffusion of pentane in a concentrated dichloromethane solution. **Anal. Calcd.** for $C_{62}H_{79}AuF_6PSb$: C, 57.82, H, 6.18. **Found:** C, 57.86; H, 6.45. **^{1}H NMR** (400 MHz, CD_2Cl_2, 25 ºC) δ: 7.77 (dt, 3H, $^3J_{HH}$ = 8.1 Hz, $^4J_{HH}$ = $^5J_{HP}$ = 1.8 Hz, H_b), 7.56 (dd, 3H, $^3J_{HP}$ = 12.4 Hz, $^4J_{HH}$ = 1.8 Hz, H_a), 7.44 (dd, 3H, $^3J_{HH}$ = 8.1 Hz, $^4J_{HP}$ = 6.0 Hz, H_c), 7.24 (d, 6H, $^3J_{HH}$ = 7.5 Hz, H_e), 6.69 (br, 6H, H_d), 3.79 (m, 2H, C_2H_4), 3.66 (m, 2H, C_2H_4), 1.27 (s, 27H,

CH_3 (^{t}Bu$_7$)), 1.27 (s, 27H, CH_3 (^{t}Bu$_3$)). **^{1}H NMR** (400 MHz, CD_2Cl_2, –70 ºC) δ: 7.70 (dt, 3H, $^3J_{HH}$ = 8.1 Hz, $^4J_{HH}$ = 1.7 Hz, H$_b$), 7.49 (dd, 3H, $^3J_{HP}$ = 12.4 Hz, $^4J_{HH}$ = 1.8 Hz, H$_a$), 7.39 (m, 6H, H$_c$, H$_d$), 7.17 (dd, 3H, $^3J_{HH}$ = 8.2 Hz, $^5J_{HH}$ = 1.8 Hz, H$_e$), 7.01 (dd, 3H, $^3J_{HH}$ = 8.1 Hz, $^5J_{HH}$ = 1.8 Hz, H$_e$), 6.09 (dt, 3H, $^3J_{HH}$ = 8.1 Hz, $^4J_{HH}$ = 1.7 Hz, H$_d$), 3.54 (m, C_2H_4), 1.18 (s, 54H, CH_3 (^{t}Bu$_3$), CH_3 (^{t}Bu$_7$)). **^{13}C{^{1}H} NMR** (100 MHz, CD_2Cl_2, 25 ºC) δ: 152.7 (C$_6$), 152.5 (d, $^3J_{CP}$ = 8 Hz, C$_2$), 143.7 (d, $^2J_{CP}$ = 16 Hz, C$_4$), 138.9 (d, $^3J_{CP}$ = 8 Hz, C$_5$), 133.9 (d, $^3J_{CP}$ = 7 Hz, CH$_a$), 133.8 (CH$_c$), 130.3 (CH$_b$), 130.1 (CH$_d$), 128.0 (d, $^1J_{CP}$ = 62 Hz, C$_1$), 126.2 (CH$_e$), 110.0 (d, $^2J_{CP}$ = 9 Hz, C_2H_4), 35.7 (C$_7$), 35.4 (C$_3$), 31.8 (C(CH$_3$)$_3$ (^{t}Bu$_7$)), 31.6 (C(CH$_3$)$_3$ (^{t}Bu$_3$)). **^{31}P{^{1}H} NMR** (162 MHz, CD_2Cl_2, 25 ºC) δ: 13.1.

Compound 5

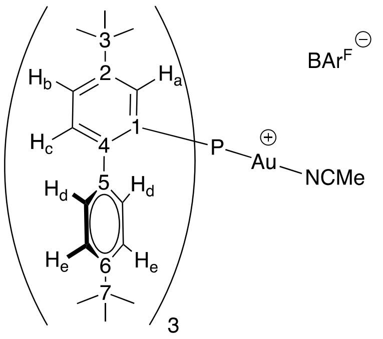

A solution of compound **2** (125 mg, 0.12 mmol), NaBAr^F (115 mg, 0.13 mmol) and MeCN (100 μL) in CH_2Cl_2 (5 mL) was stirred at 0 ºC for 1 h. The solvent was then removed under vacuum. The crude solid was dissolved in CH_2Cl_2 (10 mL) and filtered through a short pad of Celite. The solvent was removed under vacuum, thus yielding complex **5** as a white solid (165 mg, 73%). Crystals suitable for X-ray diffraction were grown by slow evaporation a dichloromethane solution of complex **5**. **Anal. Calcd.** for $C_{94}H_{90}AuBF_{24}NP$: C, 58.55; H, 4.70; N 0.73. **Found:** C, 58.71; H, 4.69; N 0.77. **^{1}H NMR** (400 MHz, CD_2Cl_2, 25 ºC) 7.77–7.70 (m, 8H, *o*-C_6H_3(3,5-CF_3)$_2$ (BAr^F)), 7.72 (dt (overlapped), 3H, $^3J_{HH}$ = 8.0 Hz, $^4J_{HH}$ = $^5J_{HP}$ = 1.6 Hz, H$_b$), 7.57 (br s, 4H, *p*-C_6H_3(3,5-CF_3)$_2$ (BAr^F)), 7.44 (dd, 3H, $^3J_{HP}$ = 12.6 Hz, $^4J_{HH}$ = 2.0 Hz, H$_a$), 7.39 (dd, 3H, $^3J_{HH}$ = 8.0 Hz, $^4J_{HP}$ = 5.7 Hz, H$_c$), 7.13 (d, 6H, $^3J_{HH}$ = 8.4 Hz, H$_e$), 6.58 (br, 6H, H$_d$), 2.10 (d, 3H, $^5J_{HP}$ = 0.8 Hz, NCCH_3), 1.26 (s, 27H, CH_3 (^{t}Bu$_3$)), 1.24 (s, 27H, CH_3 (^{t}Bu$_7$)). **^{13}C{^{1}H} NMR** (100 MHz, CD_2Cl_2, 25 ºC) δ: 162.6 (q, $^1J_{CB}$ = 50 Hz, *ipso*-C_6H_3(3,5-CF_3)$_2$ (BAr^F)), 152.1 (d, $^3J_{CP}$ = 8 Hz, C$_2$), 151.9 (C$_6$), 144.6 (d, $^2J_{CP}$ = 15 Hz, C$_4$), 138.7 (d, $^3J_{CP}$ = 8 Hz, C$_5$), 135.6 (s, *o*-C_6H_3(3,5-CF_3)$_2$ (BAr^F)), 133.3 (d, $^3J_{CP}$ = 6 Hz, CH$_a$), 133.0 (d, $^4J_{CP}$ = 9 Hz, CH$_c$), 130.0 (CH$_d$), 129.7 (q, $^2J_{CF}$ = 32 Hz, *m*-C_6H_3(3,5-CF_3)$_2$ (BAr^F)), 129.7 (CH$_b$), 127.7 (d, $^1J_{CP}$ = 67 Hz, C$_1$), 125.4 (q, $^1J_{CF}$ = 273 Hz, CF_3 (BAr^F)), 125.6 (CH$_e$), 118.3 (s, *p*-C_6H_3(3,5-CF_3)$_2$ (BAr^F)), 116.8 (d, $^3J_{CP}$ = 11 Hz, NCCH$_3$), 35.6 (C$_3$), 35.2 (C$_7$), 31.9 (C(CH$_3$)$_3$ (^{t}Bu$_7$)), 31.6 (C(CH$_3$)$_3$ (^{t}Bu$_3$)), 3.5 (NCCH$_3$). **^{31}P{^{1}H} NMR** (162 MHz, CD_2Cl_2, 25 ºC) δ: 1.6. **^{19}F{^{1}H} NMR** (376 MHz, CD_2Cl_2, 25 ºC) δ: –62.8. **^{11}B NMR** (128 MHz, CD_2Cl_2, 25 ºC) δ: –6.6.

Compound 6

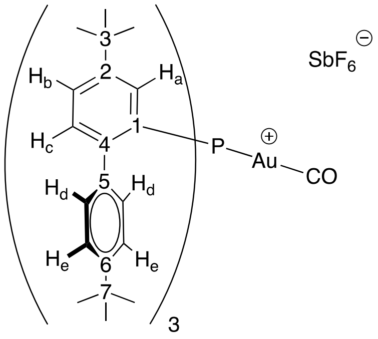

In a glovebox, a Schlenk flask was charged with silver hexafluoroantimonate (19 mg, 0.055 mmol) in dichloromethane (2 mL). Complex **2** (53 mg, 0.05 mmol) was transferred into a small glass vial and dissolved in dichloromethane (2 mL). The vial solution was loaded into a plastic syringe equipped with stainless steel needle. Outside the glovebox, the Schlenk flask was cooled down to –30 ºC. At this temperature the solution of complex **2** was added to the $AgSbF_6$ suspension while bubbling CO. The mixture was allowed to slowly warm up to room temperature. Then the mixture was filtered through a short pad of Celite to remove the silver salts affording complex **6** in quantitative NMR yield. Crystals suitable for X-ray diffraction were grown by slow diffusion of pentane into a concentrated dichloromethane solution. **^{1}H NMR** (400 MHz, CD_2Cl_2, 25 ºC) δ: 7.80 (dt, 3H, $^3J_{HH}$ = 8.1 Hz, $^4J_{HH}$ = $^5J_{HP}$ = 1.8 Hz, H_b), 7.54 (dd, 3H, $^3J_{HP}$ = 12.3 Hz, $^4J_{HH}$ = 1.8 Hz, H_a), 7.47 (dd, 3H, $^3J_{HH}$ = 8.1 Hz, $^4J_{HP}$ = 6.0 Hz, H_c), 7.27 (d, 6H, $^3J_{HH}$ = 7.9 Hz, H_e), 6.65 (br, 6H, H_d), 1.29 (s, 27H, CH_3 (^{t}Bu$_3$)), 1.26 (s, 27H, CH_3 (^{t}Bu$_7$)). **^{13}C{^{1}H} NMR** (100 MHz, CD_2Cl_2, 25 ºC) δ: 182.6 (d, $^2J_{CP}$ = 110 Hz, CO), 153.0 (C_6), 152.8 (d, $^3J_{CP}$ = 8 Hz, C_2), 143.9 (d, $^2J_{CP}$ = 16 Hz, C_4), 138.5 (d, $^3J_{CP}$ = 8 Hz, C_5), 133.4 (d, $^3J_{CP}$ = 6 Hz, CH_a), 133.1 (d, $^4J_{CP}$ = 9 Hz, CH_c), 130.7 (CH_b), 130.5 (CH_d), 126.6 (CH_e), 126.5 (d, $^1J_{CP}$ = 66 Hz, C_1), 35.7 (C_3), 35.4 (C_7), 31.7 (C(CH$_3$)$_3$ (^{t}Bu$_7$)), 31.6 (C(CH$_3$)$_3$ (^{t}Bu$_3$)). **^{31}P{^{1}H} NMR** (162 MHz, CD_2Cl_2, 25 ºC) δ: 6.9.

2. NMR spectroscopic experiments

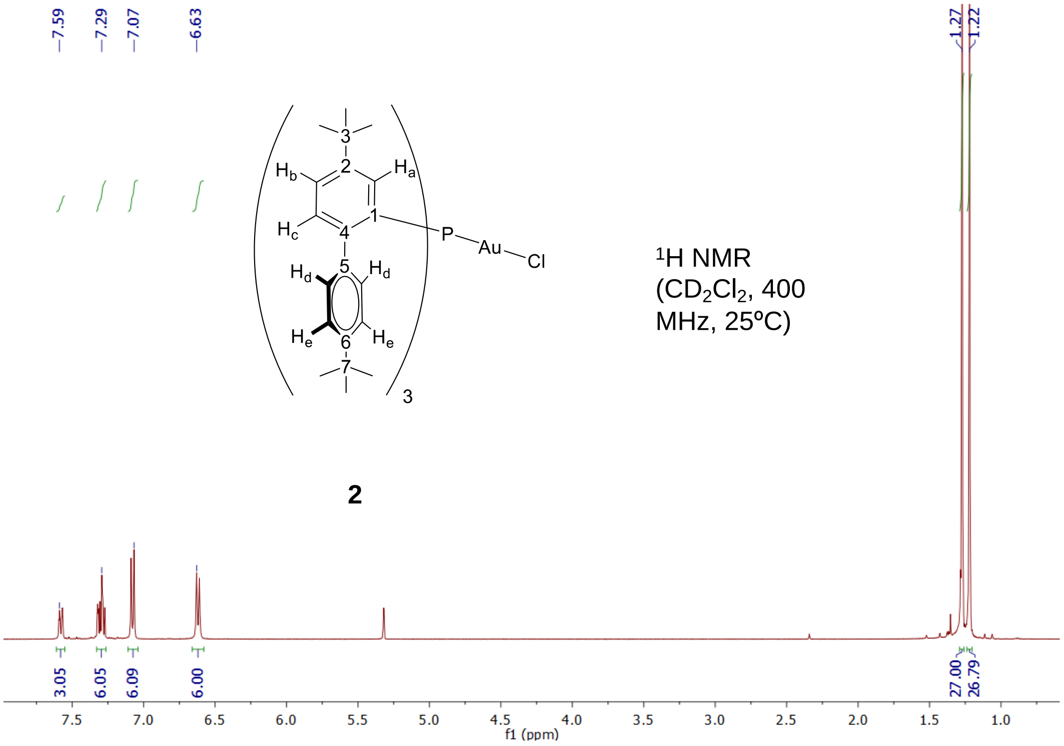

Figure S1. ^1H NMR of complex **2**.

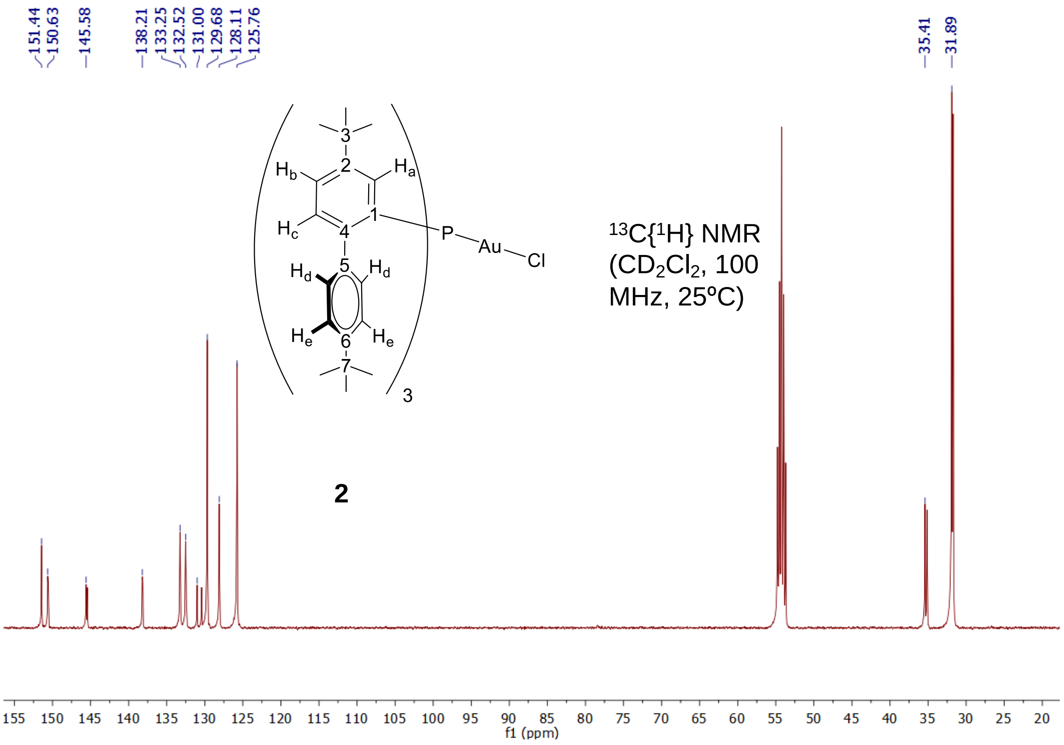

Figure S2. $^{13}C\{^1H\}$ NMR of complex **2**.

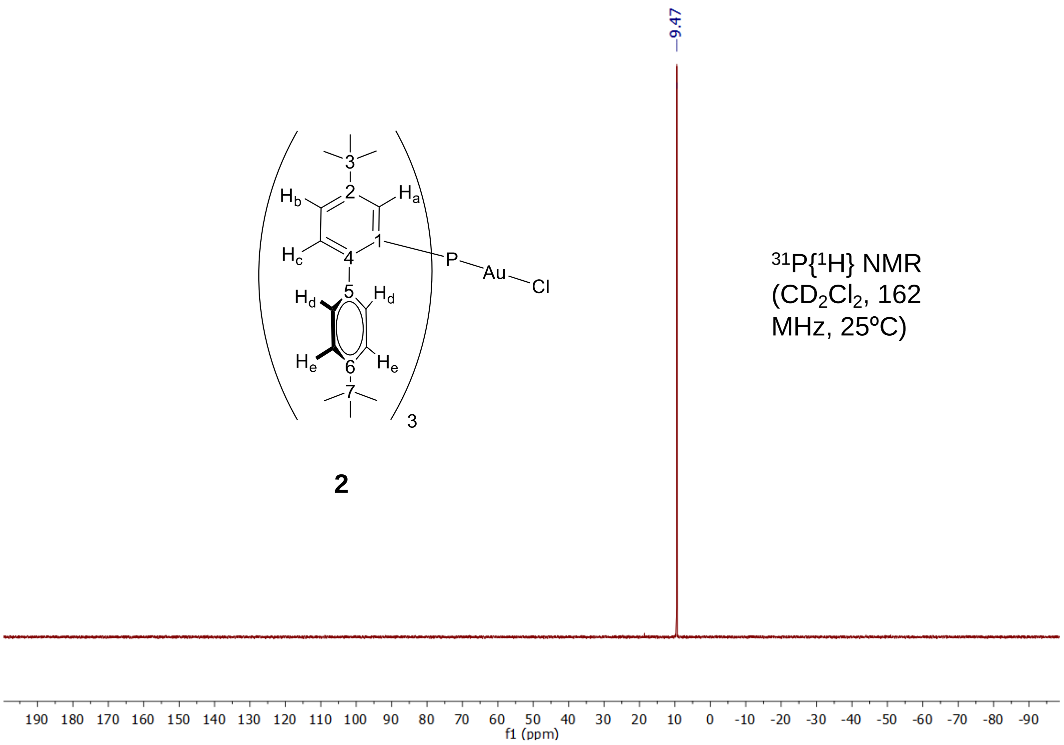

Figure S3. $^{31}P\{^{1}H\}$ NMR of complex **2**.

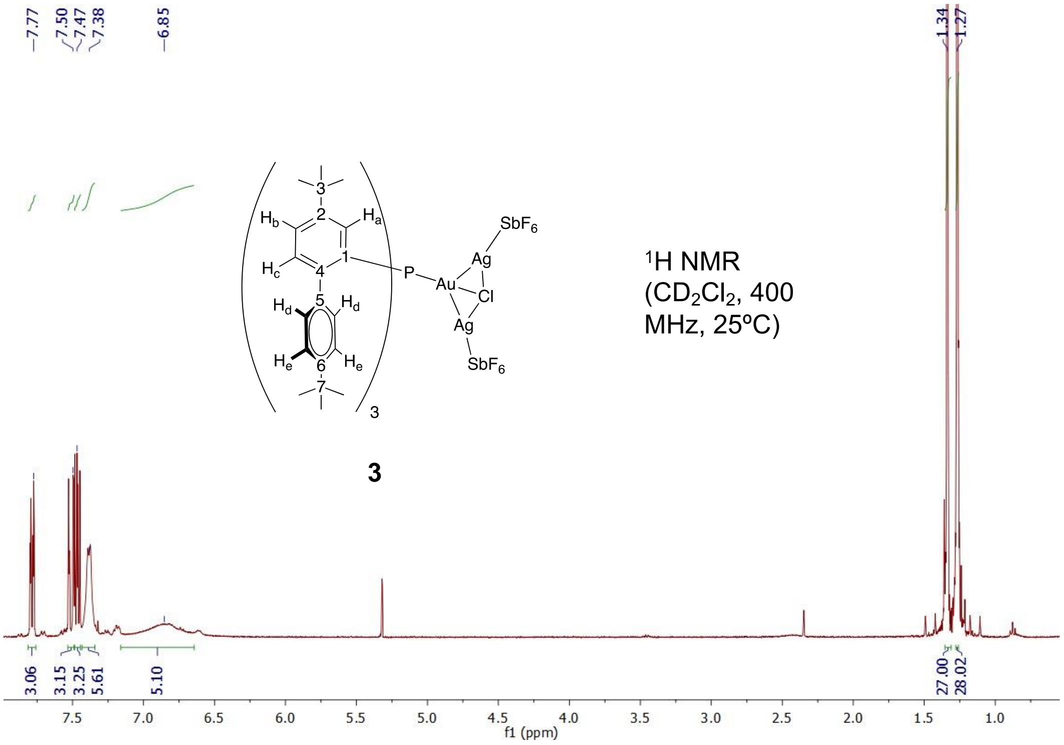

Figure S4. ^{1}H NMR of complex **3**.

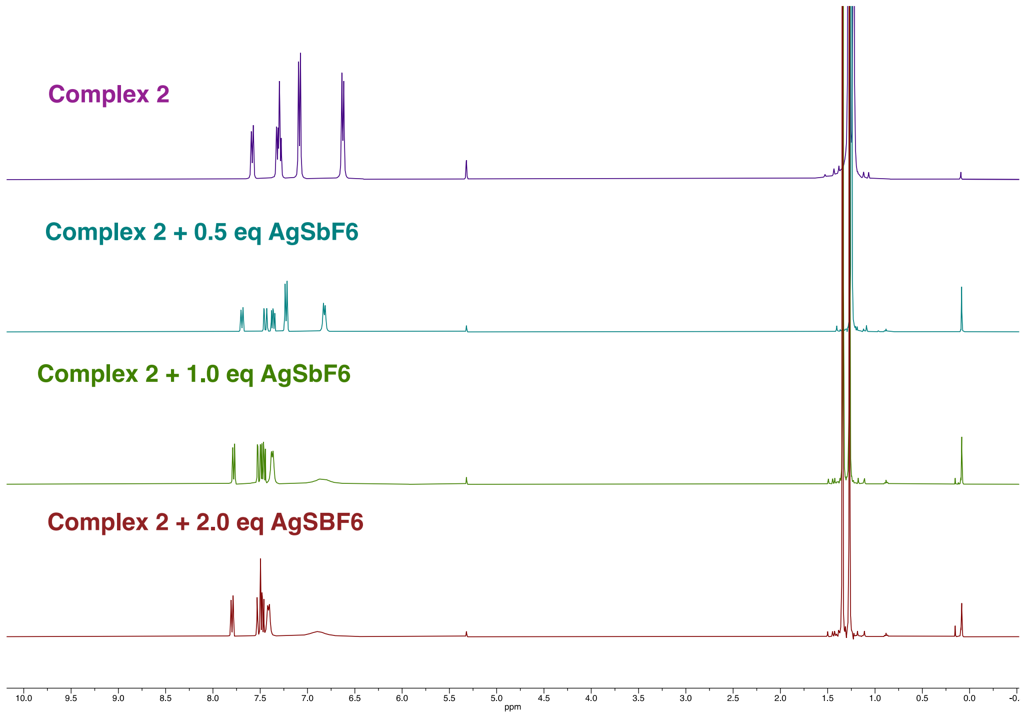

Figure S5. ^{1}H NMR of consecutive additions of $AgSbF_6$ to complex **2**.

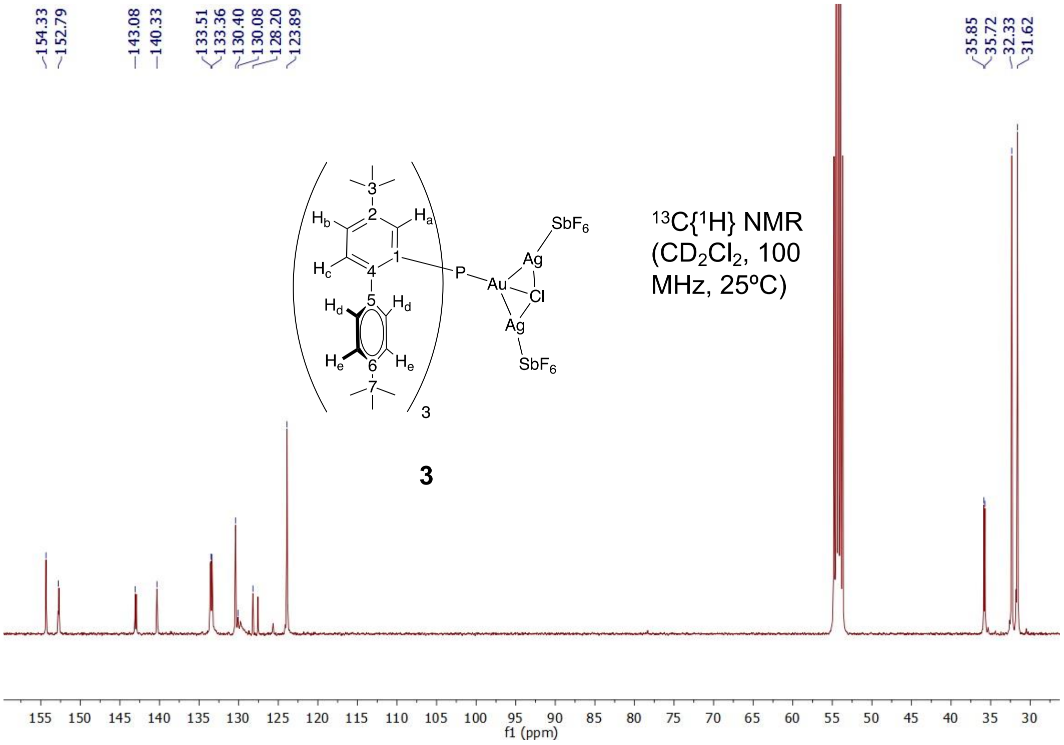

Figure S6. $^{13}C\{^{1}H\}$ NMR of complex **3**.

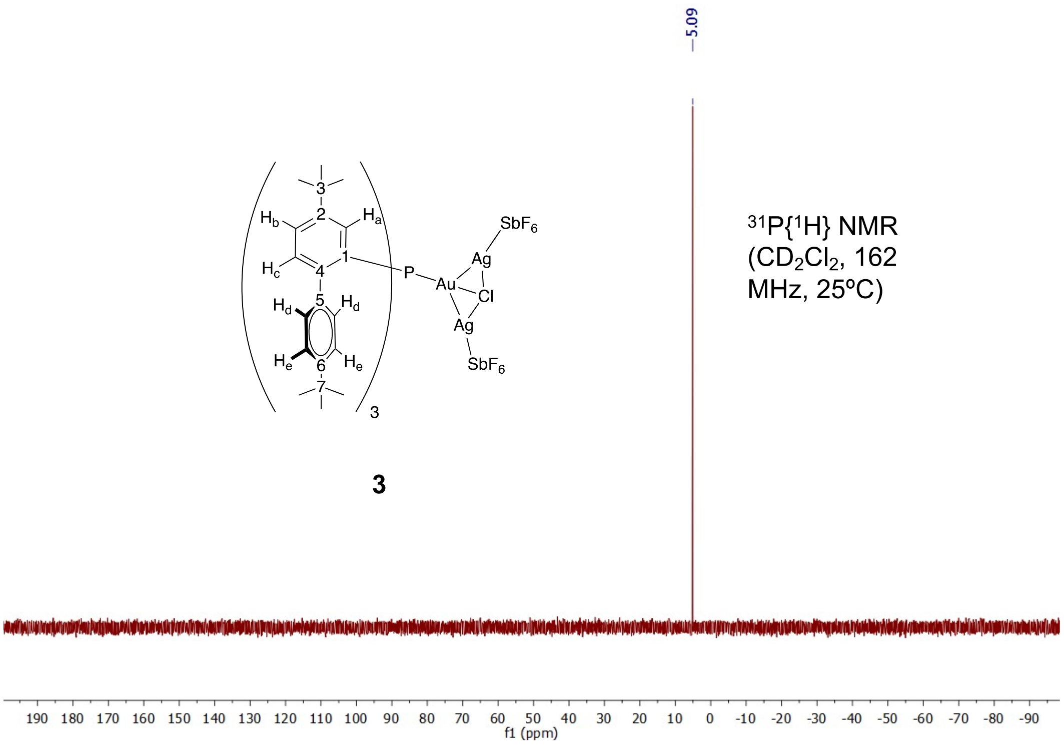

Figure S7. $^{31}P\{^1H\}$ NMR of complex **3**.

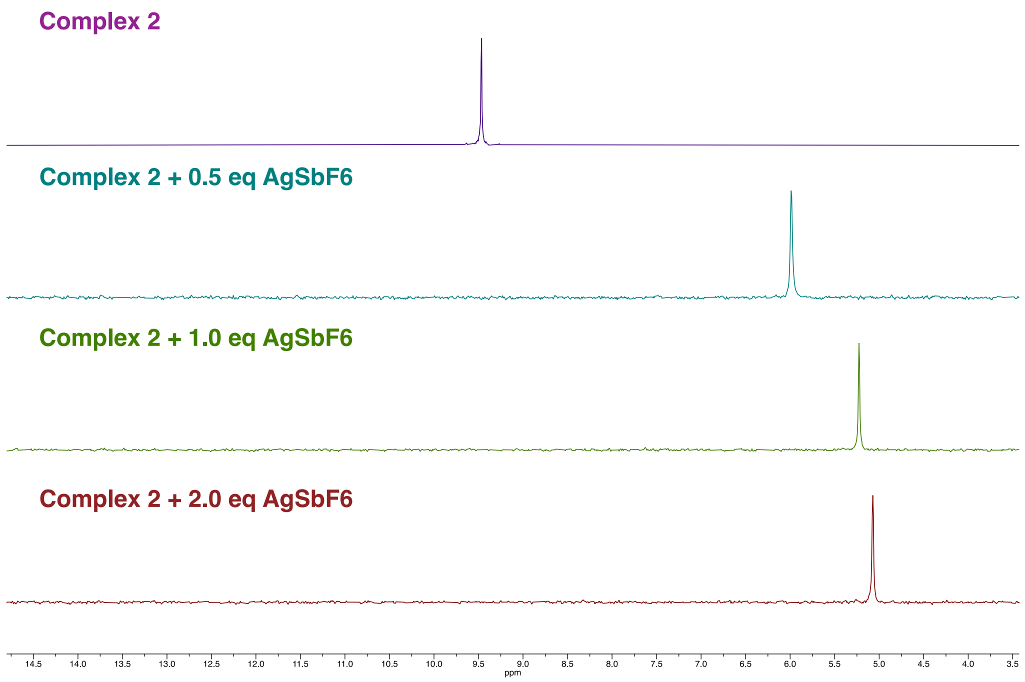

Figure S8. Stacked $^{31}P\{^1H\}$NMR of consecutive additions of $AgSbF_6$ to complex **2**.

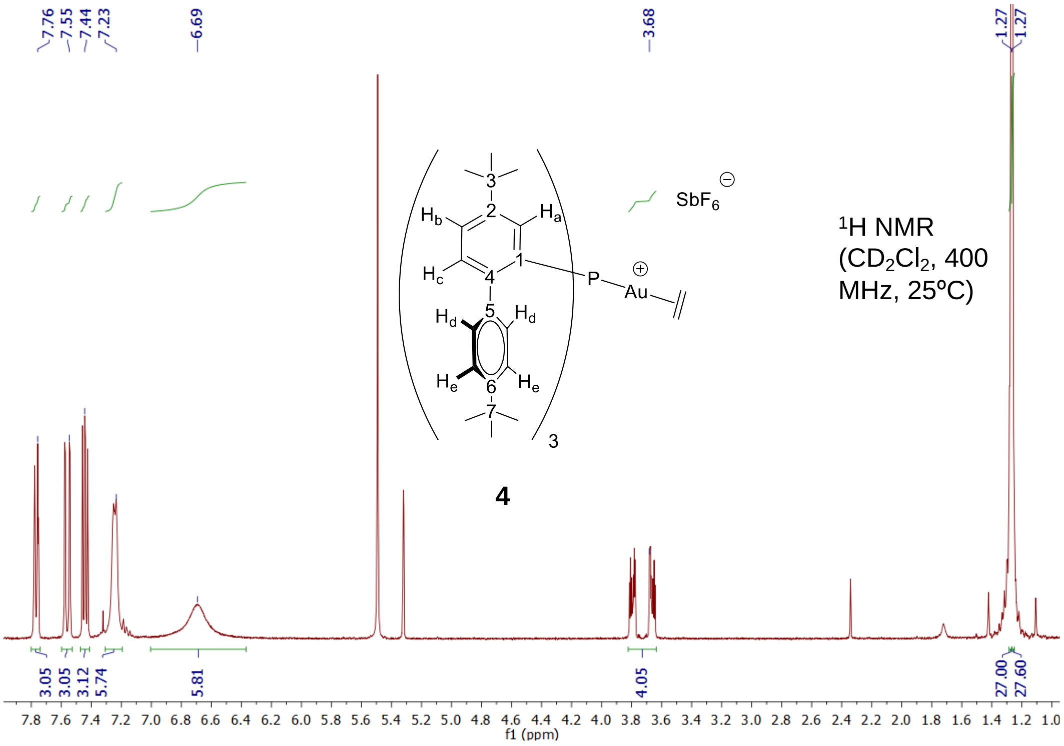

Figure S9. ^{1}H NMR of complex **4** (25ºC).

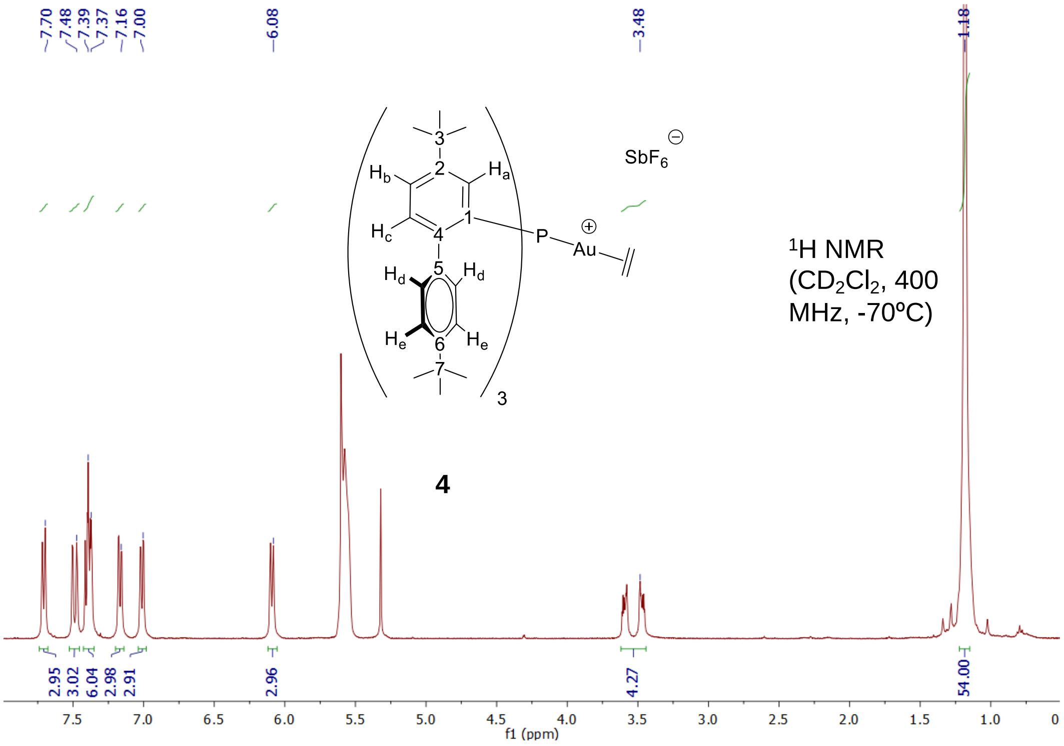

Figure S10. ^{1}H NMR of complex **4** (–70 ºC).

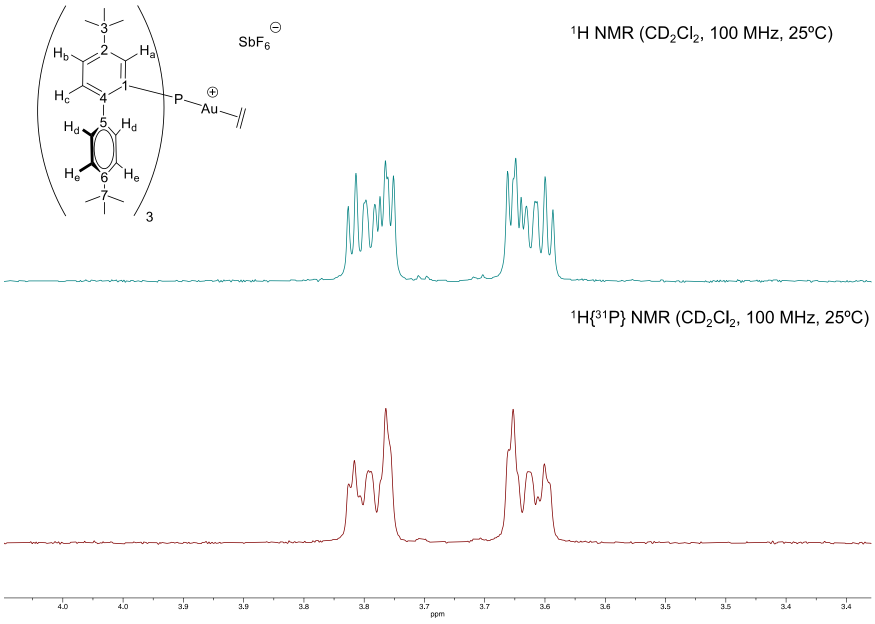

Figure S11. Stacked ^{1}H NMR and ^{1}H{^{31}P} NMR spectra of complex **4** in the region where coordinated ethylene resonates.

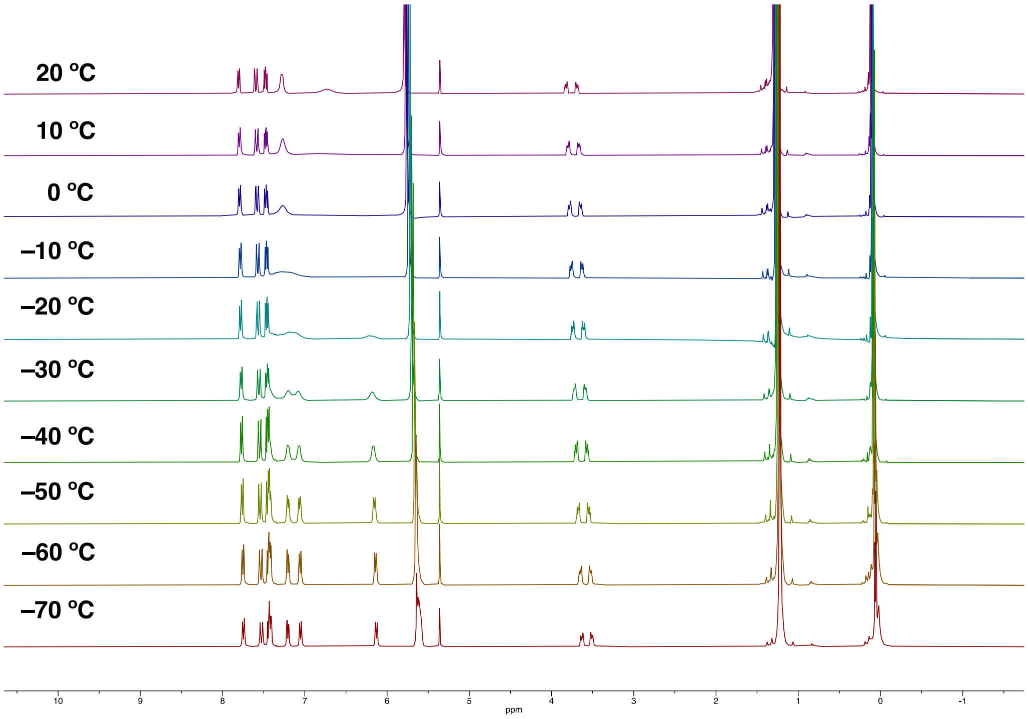

Figure S12. Variable temperature ^{1}H NMR spectra of complex **4** in CD_2Cl_2 from –70 ºC to 20 ºC with 10 ºC increments.

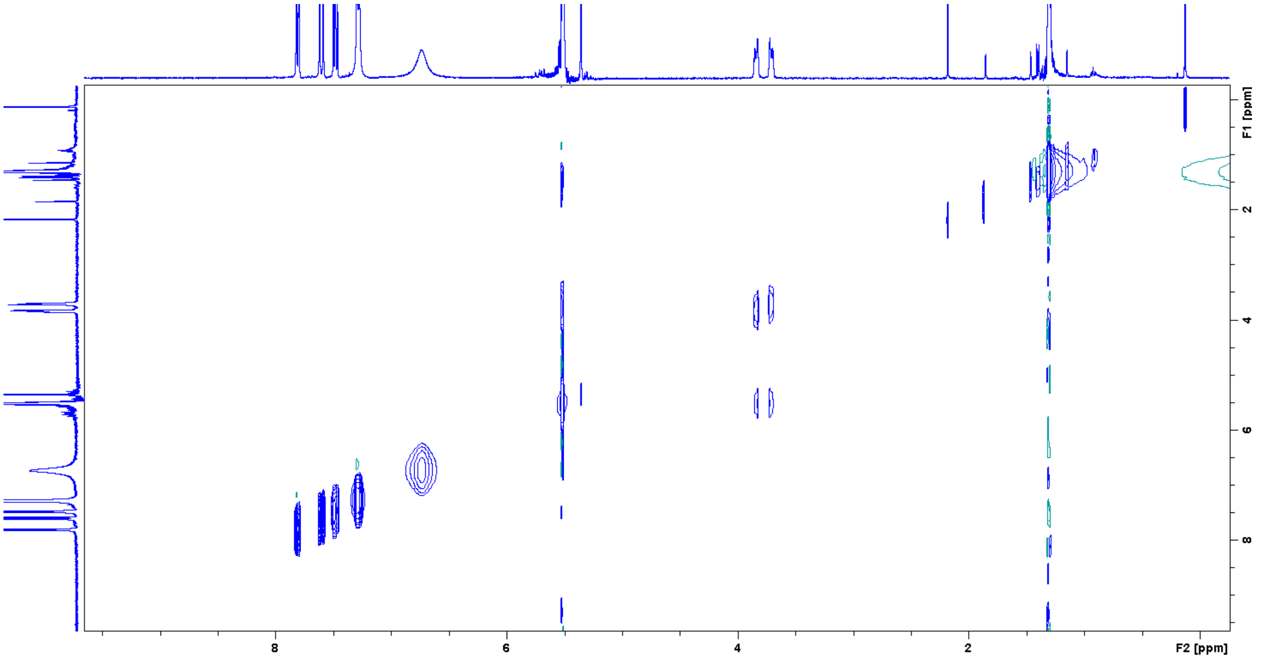

Figure S13. NOESY spectrum of **4** recorded at 30 ºC. Exchange cross-peaks were detected for signals belonging to coordinated and free ethylene.

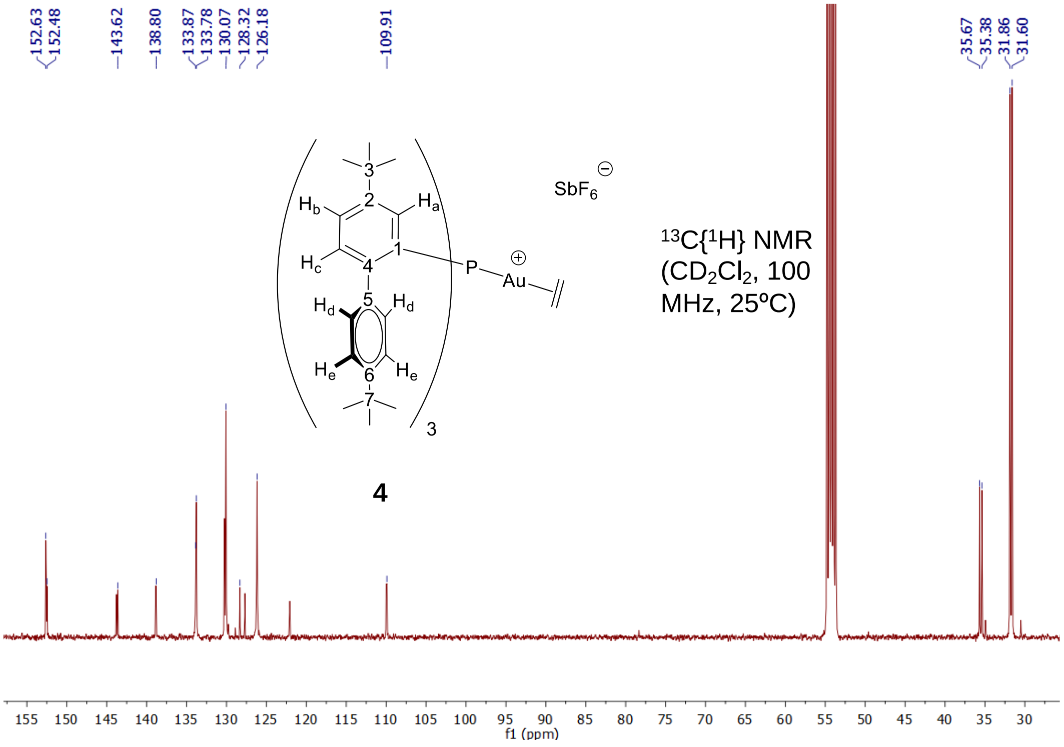

Figure S14. $^{13}C\{^1H\}$ NMR of complex **4**.

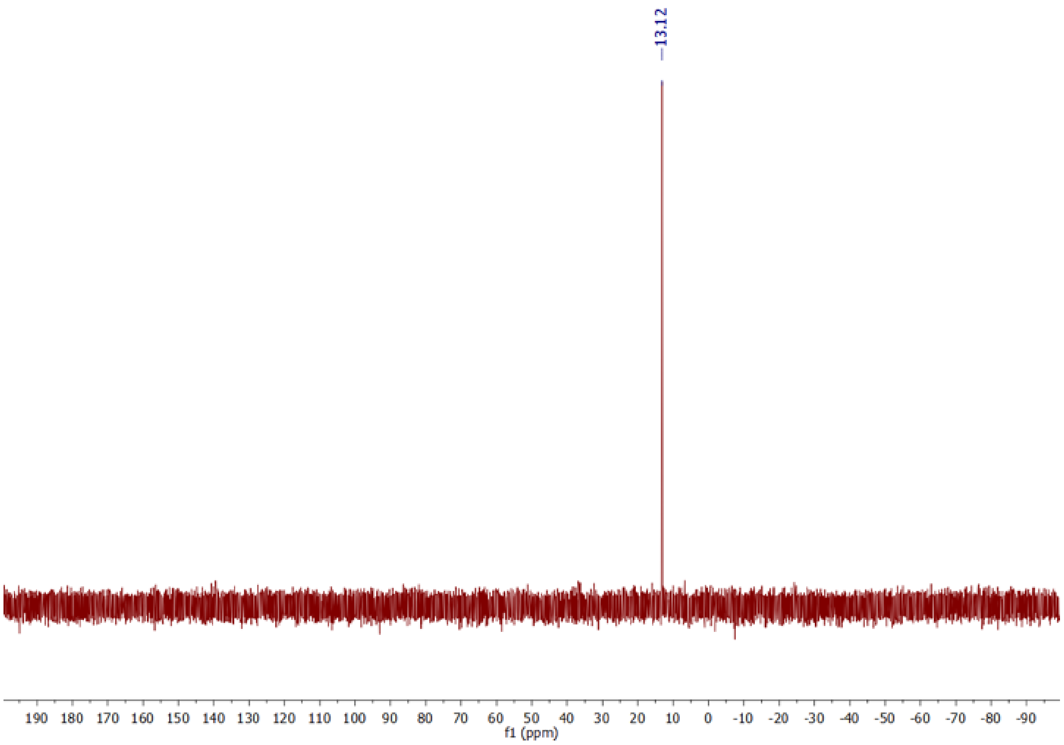

Figure S15. $^{31}P\{^{1}H\}$ NMR of complex **4**.

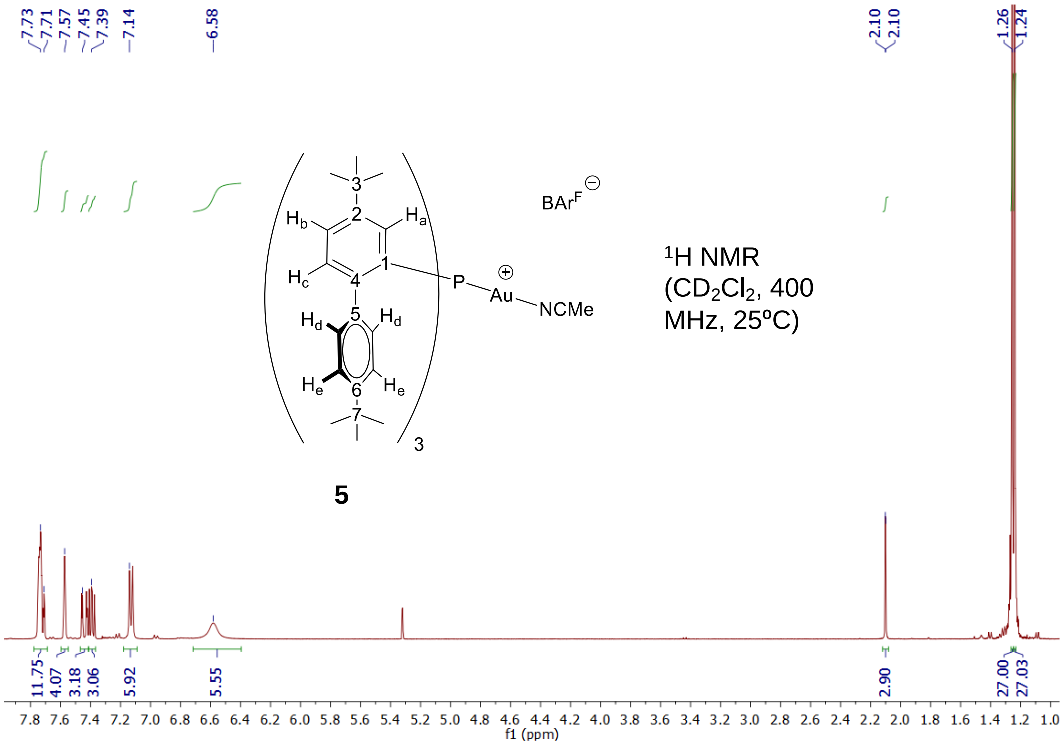

Figure S16. ^{1}H NMR of complex **5**.

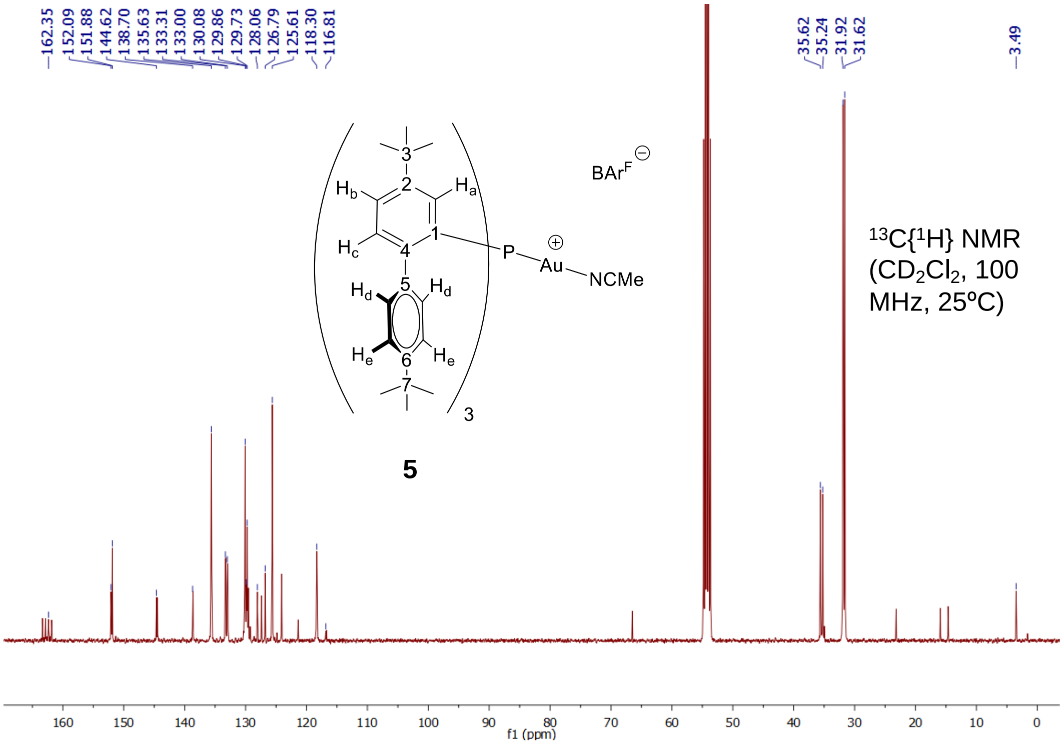

Figure S17. $^{13}C\{^1H\}$ NMR of complex **5**.

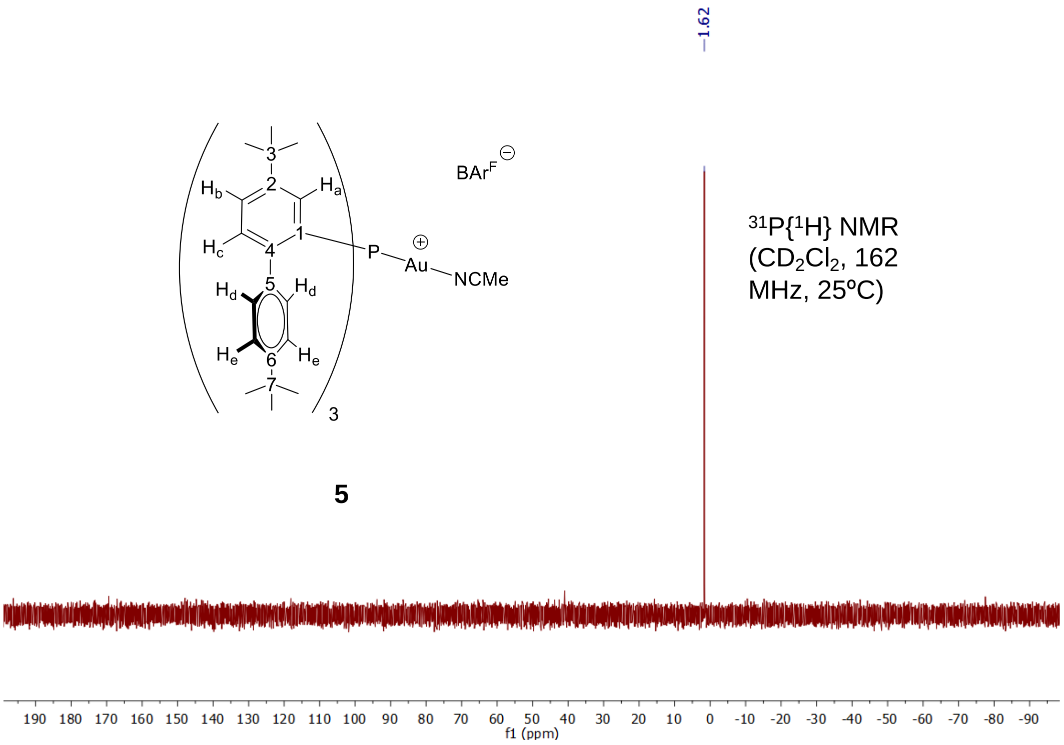

Figure S18. $^{31}P\{^1H\}$ NMR of complex **5**.

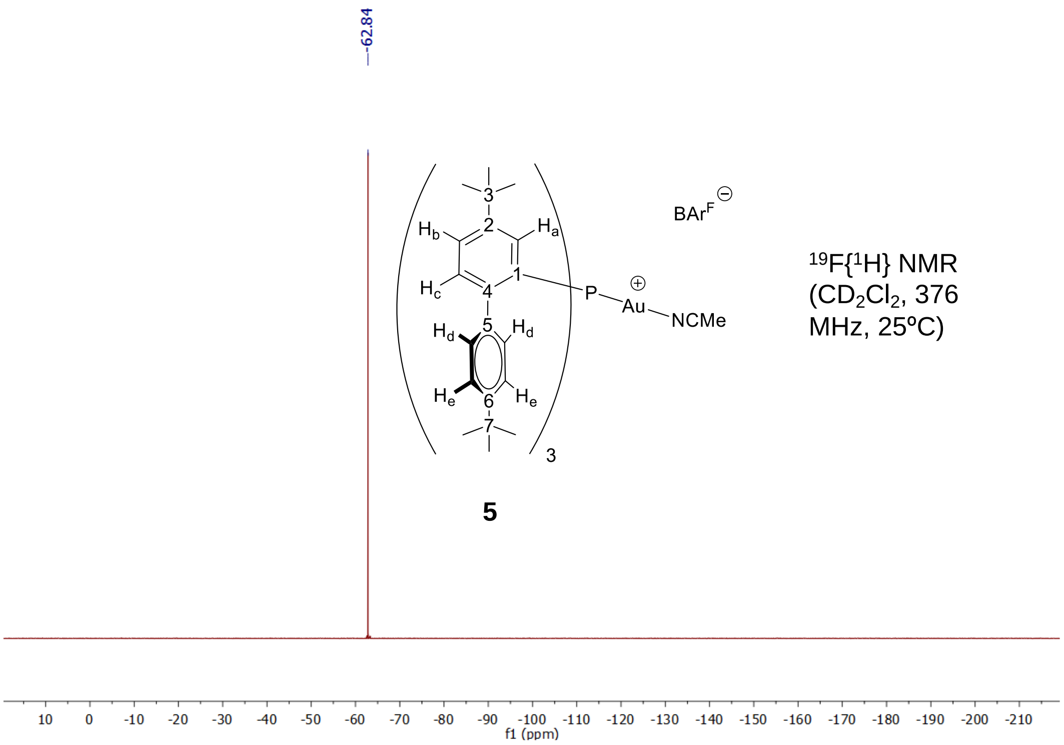

Figure S19. ^{19}F{^{1}H} NMR of complex **5**.

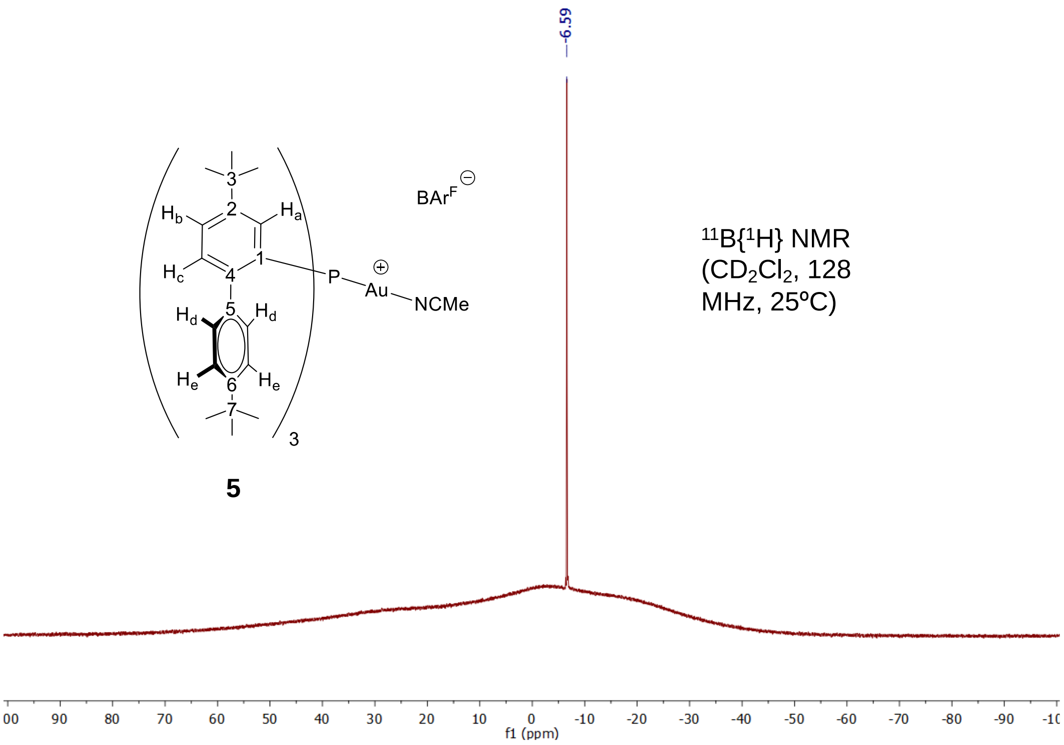

Figure S20. ^{11}B{^{1}H} NMR of complex **5**.

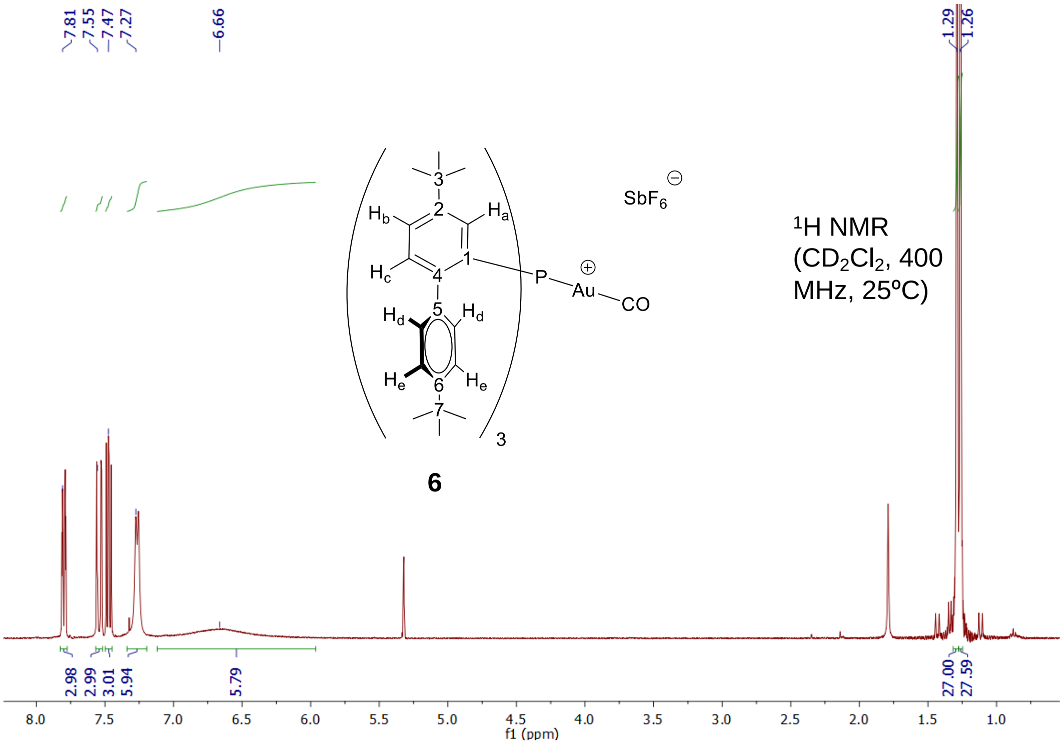

Figure S21. ^{1}H NMR of complex **6**.

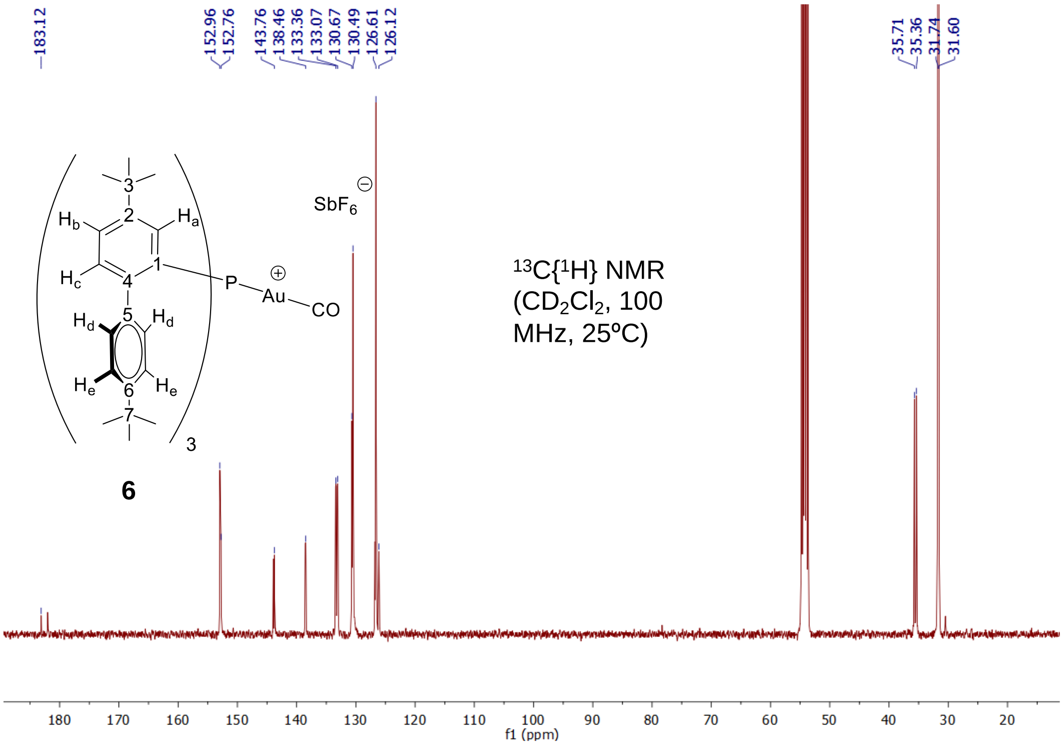

Figure S22. ^{13}C{^{1}H} NMR of complex **6**.

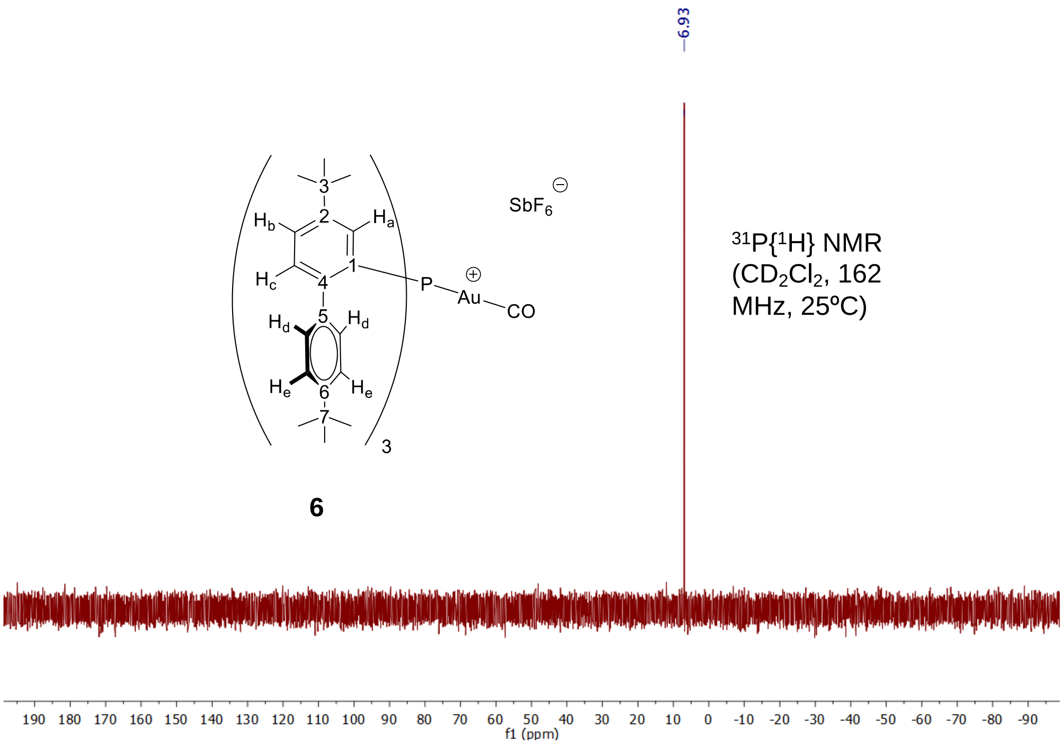

Figure S23. $^{31}P\{^{1}H\}$ NMR of complex **6**.

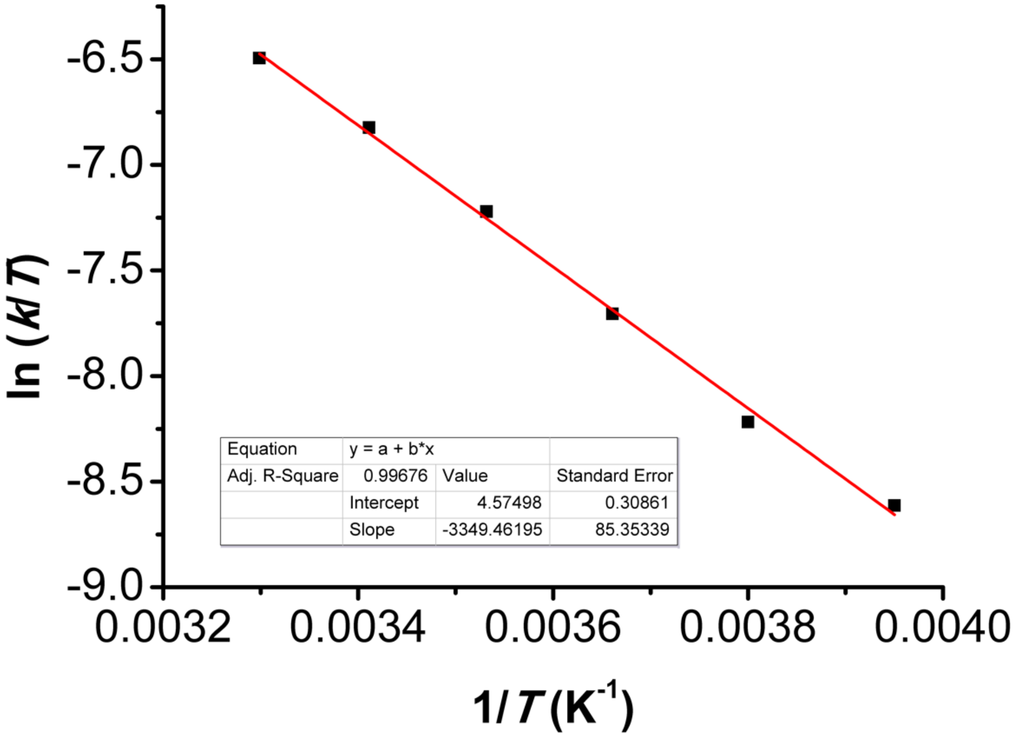

Figure S24. Eyring plot for the exchange of bound and free ethylene in complex **4** from −20 to 30 ºC.

Exchange experiments between complexes 4, 5 and 6:

Adding MeCN or CO in excess to a solution of complex **4** quantitatively afforded the displacement of the coordinated ethylene molecule: $^{31}P\{^{1}H\}$ NMR monitoring revealed the appearance of new signals at 1.3 ppm and 6.9 ppm, corresponding to the gold(I)–acetonitrile (**5**) and carbonyl (**6**) adducts, respectively. In addition, ^{1}H NMR spectroscopy revealed the presence of free ethylene at 5.40 ppm, and a new doublet at 2.10 ppm ($^{5}J_{HP}$ = 0.8 Hz) due to the coordinated MeCN molecule in 5. Despite the apparent lability of the gold–ethylene moiety, reversible formation of 4 was achieved by adding excess ethylene to dichloromethane solutions of either **5** or **6**. Exchange experiments in CD_2Cl_2 between complexes **4**, **5** an **6** were performed as indicated:

In a Young NMR tube complex **2** (10 mg, 0.01 mmol) and $AgSbF_6$ (7 mg, 0.02 mmol) were dissolved in CD_2Cl_2 (0.5 mL). The tube was charged with 1 bar of a mixture of ethylene (0.5 bar) and CO (0.5 bar) and intensely shaken for half an hour. The reaction was monitored by ^{1}H and ^{31}P NMR spectroscopy until equilibrium conditions were reached (Figure S25).

Similarly, a solution of complex **5** (19 mg, 0.01 mmol) in CD_2Cl_2 (0.5 mL) was charged either with 1 bar of ethylene or 1 bar of CO and intensely shaken for half an hour. The reactions were both monitored by ^{1}H and ^{31}P NMR spectroscopy until equilibrium conditions were reached (Figure S25).

The equilibrium constants calculated accordingly:

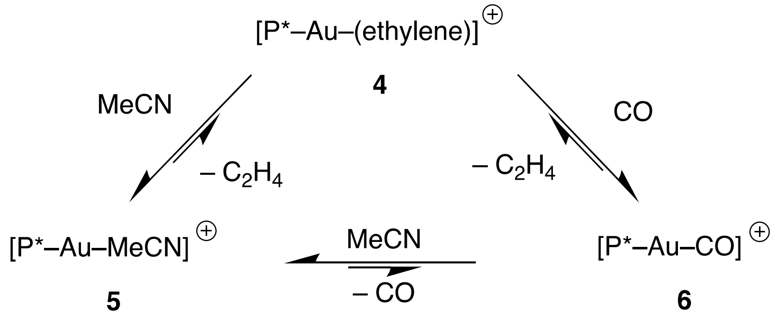

$$K_{eq1} = [\mathbf{4}][\mathrm{MeCN}]/[\mathbf{5}][\mathrm{C_2H_4}] = 0.0018$$

$$K_{eq2} = [\mathbf{6}][\mathrm{MeCN}]/[\mathbf{5}][\mathrm{CO}] = 0.18$$

$$K_{eq3} = [\mathbf{6}][\mathrm{C_2H_4}]/[\mathbf{5}][\mathrm{CO}] = 113$$

Scheme S1. Reaction equilibria between complexes **4**, **5**, and **6** (P* = tris-2-(4,4’-di-tert-butylbiphenylyl)phosphine) and equilibrium constants obtained from NMR studies.[3]

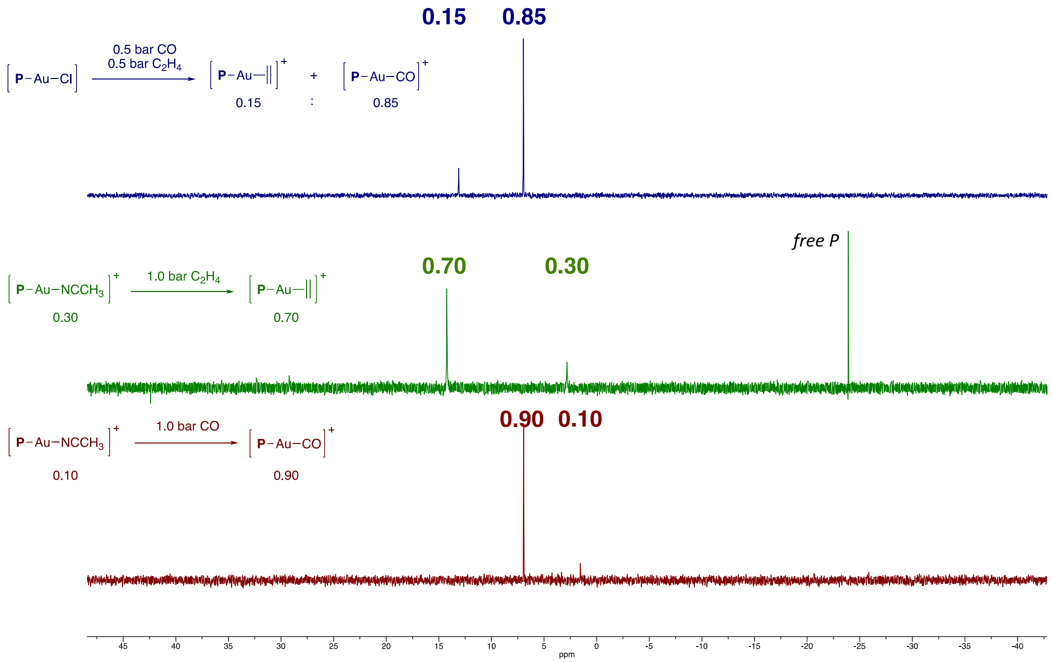

Figure S25. $^{31}P\{^{1}H\}$ NMR of exchange experiments between complexes **4**, **5** and **6** when equilibrium conditions are reached.

3. IR spectrum of complex 6

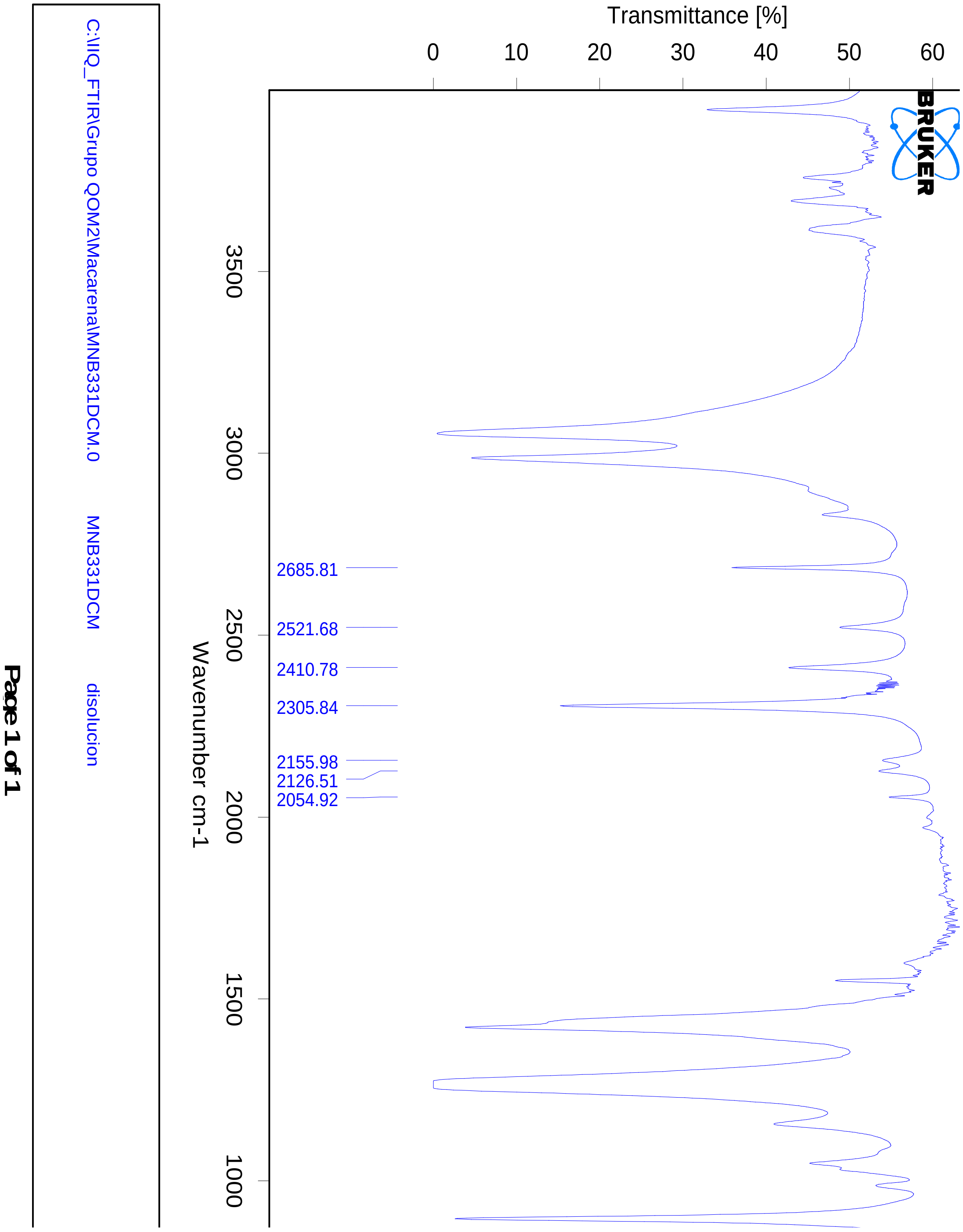

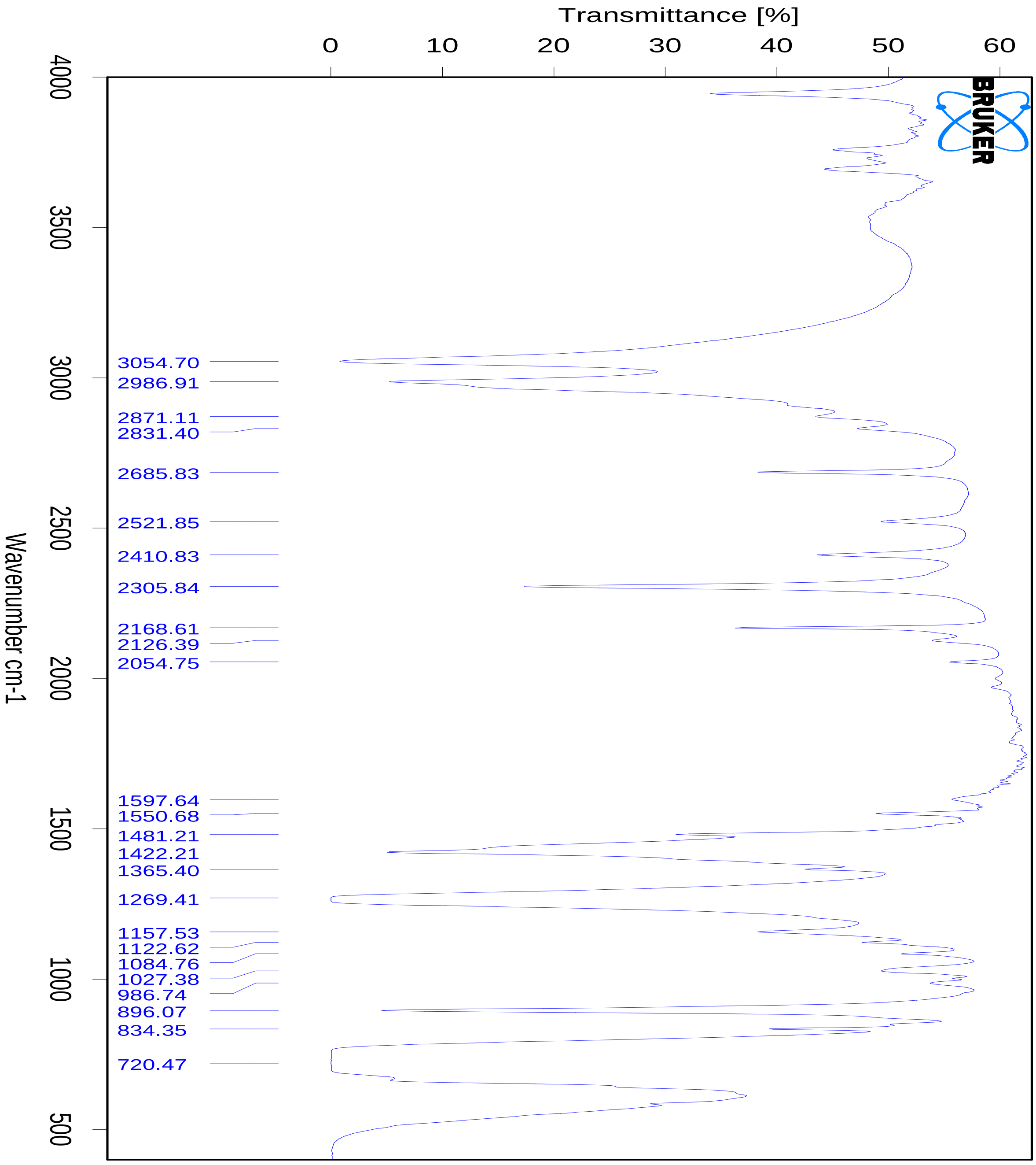

Figure S26. IR spectrum of dichloromethane (first) and complex **6** in dichloromethane (after).

4. Crystal structure determinations

Crystallographic details. Low-temperature diffraction data were collected on a Bruker APEX-II CCD diffractometer (**2**) or on a D8 Quest APEX-III single crystal diffractometer with a Photon III detector and a IμS 3.0 microfocus X-ray source (**3**, **4**, **4'**, **5**, **6**) at the Instituto de Investigaciones Químicas, Sevilla. Data were collected by means of ω and φ scans using monochromatic radiation λ(Mo Kα1) = 0.71073 Å. The diffraction images collected were processed and scaled using APEX-II or APEX-III software, respectively. The structures were solved with SHELXT and was refined against F^2 on all data by full-matrix least squares with SHELXL.[4] All non-hydrogen atoms were refined anisotropically. Hydrogen atoms were included in the model at geometrically calculated positions and refined using a riding model, unless otherwise noted. In compounds **4** and **4**' the hydrogens associated to the bound ethylene ligands were calculated using the AFIX 23 command. The isotropic displacement parameters of all hydrogen atoms were fixed to 1.2 times the U value of the atoms to which they are linked (1.5 times for methyl groups). The nature of the phosphine ligand, containing six *tert*-butyl groups, lead to several features that are common to most measured structured. First, the highly disordered tert-butyl groups along with the higher libration of their terminal methyl groups led us to model the disorder for the worse behaved fragments, while restraints were applied to the corresponding ADPs. Besides, the presence of these groups generates voids during the packing that is occupied by solvent molecules. In four of the six reported structures we used the program SQUEEZE to compensate for the contribution of disordered solvents, which account for 14 pentane molecules (**3**), 4 pentane molecules (**4**), 6 pentane molecules (**5**) and 4 pentane molecules (**6**), in the unit cell. In compound **3** Compound **3** was refined as an inversion twin. We could not obtain good quality crystals for compound **5** despite many attempts. However, we could grow crystals of enough quality for X-ray diffraction studies using $NaBAr_F$ (BAr_F^- = $[B(C_6H_2\text{-}3,5\text{-}(CF_3)_2)_4]^-$) instead of $AgSbF_6$ as chloride abstractor. Thus, the reported crystal structure contains the latter anion.

The full numbering scheme of all the reported structures can be found in the full details of the X-ray structure determination (CIF), which is included as Supporting Information.

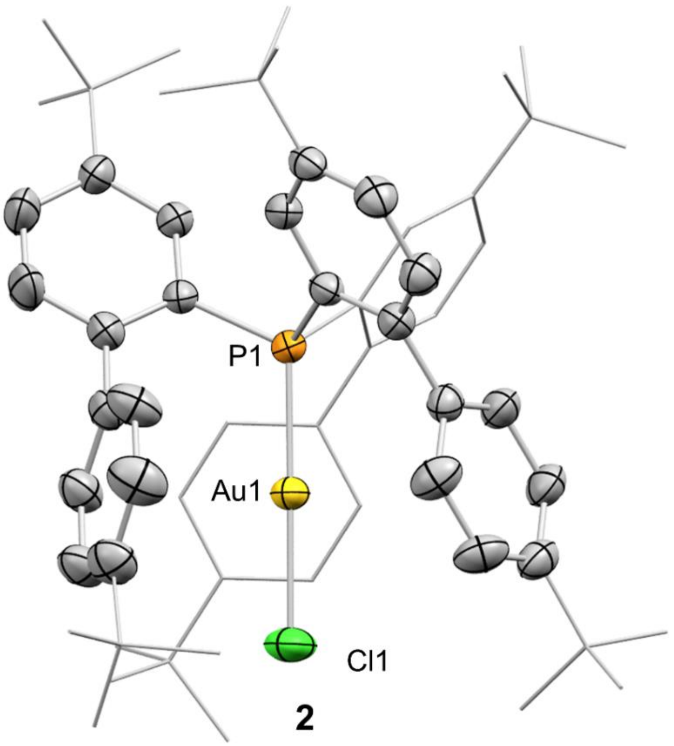

2

Figure S27. ORTEP diagram of compound **2**. Solvent molecules and hydrogen atoms are excluded for clarity, while *tert*-butyl groups and one biaryl fragment are represented in wireframe format. Thermal ellipsoids are set at 50% probability.

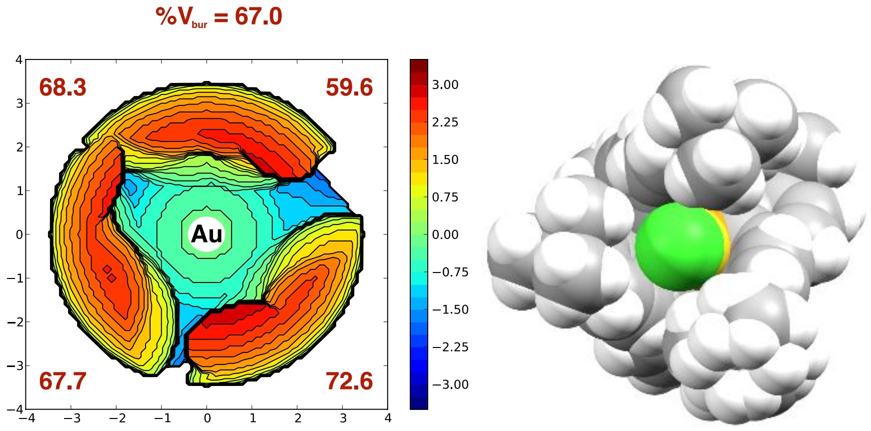

Figure S28. (Left) Steric map and calculated % VBur of the phosphine ligand **1** in complex **2**, derived from the X-ray structure. The % VBur of each quadrant are also indicated; (right) Space-fill representation of the X-ray diffraction structure of **2** along the same orientation than in the steric map.

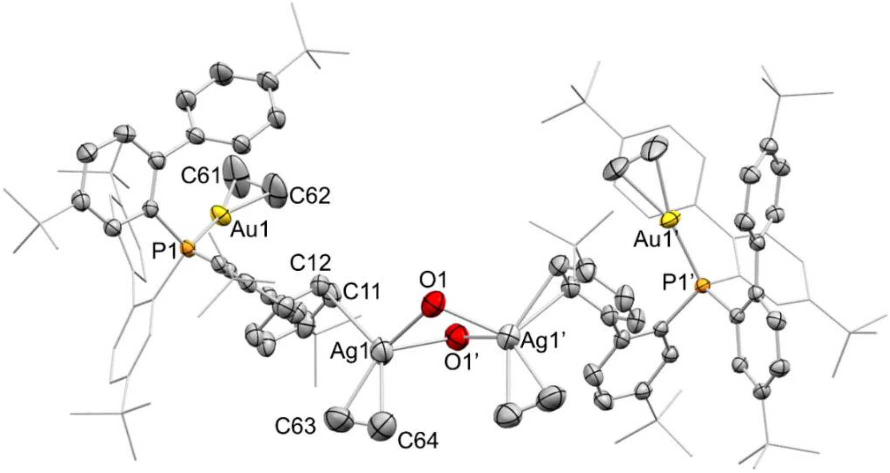

4’

Figure S29. ORTEP diagram of compound **4’**. Counteranions, solvent molecules and hydrogen atoms are excluded for clarity, while *tert*-butyl groups and one biaryl fragment are represented in wireframe format. Thermal ellipsoids are set at 50% probability. The structure has a crystallographically-imposed twofold symmetry and, as such, atom labels with an additional prime character are at equivalent positions (1-x, y, 3/2-z). Selected bond lengths (Å) and angles (°): P1–Au1, 2.2896(10); C61–C62, 1.263(10); Au1–C61, 2.216(6); Au1–C62, 2.235(6); Ag1–C11, 2.543(5); Ag1–C12, 2.612(5); Ag1–O1, 2.433(4); Ag1–C63, 2.366(7); Ag1–C64, 2.361(8); P1–Au1–C61, 166.9(2); P1–Au1–C62, 159.8(2).

The molecular structure of complexes **5** (Figure S30) and **6** (Figure 2) was further confirmed by X-ray diffraction studies. The two complexes present similar molecular structures with the gold centre adopting a linear geometry with P–Au–N and P–Au–C angles of 178.96(10)° and 176.6(2)°, respectively. The C–O bond length of 1.075(9) Å in complex **6**, shortened with respect to free CO (d_{CO} = 1.13 Å), is in agreement with those described in the literature for the very few related gold(I)–carbonyl compounds. Similarly to **4**, complex **6** co-crystalized with a silver cation, which linked two gold(I)–CO adducts by coordinating to two ortho-aryl groups of two different phosphine ligands to form a 1D-polymeric structure. Once more, the presence of silver in the bulk sample of **6** is minimal: the reported solid-state structure derives from the considerably improved capacity of silver to provide crystals of good quality. In fact, we have measured other silver-free structures of **6** of noticeably poorer crystalline quality that exhibit virtually identical geometrical parameters for the gold–carbonyl fragment.

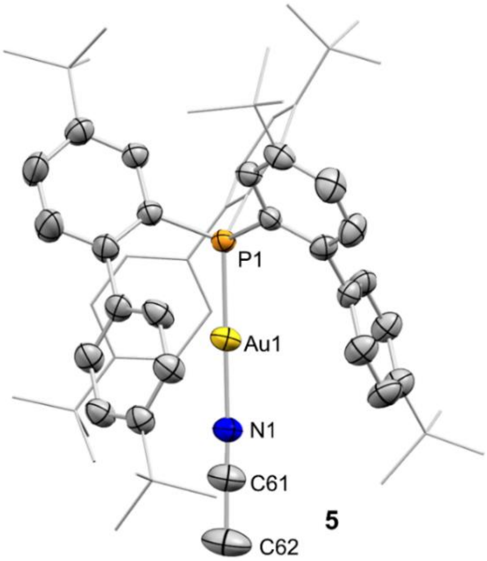

5

Figure S30. ORTEP diagram of compound **5**. Counteranion, solvent molecules and hydrogen atoms are excluded for clarity, while *tert*-butyl groups and one biaryl fragment are represented in wireframe format. Thermal ellipsoids are set at 50% probability.

Table S1. Crystal data and structure refinement for compounds **2**, **3** and **4**.

	**2**	**3**	**4**
Formula	$C_{60}H_{75}AuClP$	$C_{121}H_{152}Ag_4Au_2Cl_4F_{24}P_2Sb_4$	$C_{62}H_{79}AuF_6PSb$
Fw	1059.58	3578.57	1287.93
cryst.size, mm	0.20 × 0.17 × 0.12	0.21 × 0.07 × 0.06	0.13 × 0.12 × 0.06
crystal system	Triclinic	Orthorhombic	Monoclinic
space group	*P-1*	$Pca2_1$	$P2_1/c$
a, Å	12.6896 (7)	26.3590 (7)	13.6101 (3)
b, Å	13.4566 (7)	20.0663 (6)	51.1209 (11)
c, Å	16.6155 (5)	26.8991 (7)	18.3962 (4)
α, deg	81.539 (1)	90	90
β, deg	87.656 (1)	90	98.902 (1)
γ, deg	83.232 (1)	90	90
V, Å^3	2786.0 (2)	14227.7 (7)	12645.2 (5)
T, K	193	193	193
Z	2	4	8
ρ_{calc}, g cm^{-3}	1.263	1.671	1.353
μ, mm^{-1} (MoKα)	2.75	3.47	2.82
F(000)	1092	6968	5200
absorption corrections	multi-scan, 0.51–0.75	multi-scan, 0.58–0.75	multi-scan, 0.65–0.75
θ range, deg	2.9 – 29.5	2.0 – 26.4	1.9 – 27.1
no. of rflns measd	73945	105201	213527
R_{int}	0.036	0.068	0.040
no. of rflns unique	15328	27683	27907
no. of params / restraints	617 / 0	1494 / 467	1314 / 54
R_1 ($I > 2\sigma(I)$) [a]	0.032	0.046	0.049
R_1 (all data)	0.041	0.074	0.055
wR_2 ($I > 2\sigma(I)$)	0.087	0.109	0.121
wR_2 (all data)	0.093	0.126	0.125
Diff.Fourier.peaks min/max, eÅ^{-3}	-0.92 / 1.12	-1.22 / 2.83	-2.24 / 2.85
CCDC number	2078527	2078524	2078528

Table S2. Crystal data and structure refinement for compounds **4'**, **5** and **6**.

	**4'**	**5**	**6**
formula	$C_{128}H_{170}Ag_2Au_2F_{24}O_2P_2Sb_4$	$C_{94}H_{90}AuBF_{24}NP$	$C_{66}H_{87}AgAuF_{12}OPSb_2$
Fw	3355.24	1928.41	1703.66
cryst.size, mm	0.16 × 0.15 × 0.10	0.12 × 0.11 × 0.05	0.19 × 0.14 × 0.14
crystal system	Monoclinic	Triclinic	Triclinic
space group	$P2_1/c$	*P-1*	*P-1*
a, Å	23.1321 (5)	13.2698 (12)	13.5895 (9)
b, Å	13.8142 (3)	19.2125 (18)	16.7018 (12)
c, Å	23.2907 (6)	22.968 (2)	18.5193 (13)
α, deg	90	109.011 (3)	112.837 (3)
β, deg	112.711 (1)	90.297 (3)	□ = 102.452 (2)
γ, deg	90	106.613 (3)	□ = 94.349 (3)
V, Å^3	6865.5 (3)	5273.8 (8)	*V* = 3722.8 (5)
T, K	193	193	193
Z	2	2	2
ρ_{calc}, g cm^{-3}	1.622	1.214	1.520
μ, mm^{-1} (MoKα)	3.28	1.49	3.03
F(000)	3308	1952	1684
absorption corrections	multi-scan, 0.52 – 0.75	multi-scan, 0.51 – 0.75	multi-scan, 0.51 – 0.75
θ range, deg	1.9 – 28.3	1.9 – 28.3	2.0 – 28.4
no. of rflns measd	61002	136603	120540
R_{int}	0.033	0.065	0.075
no. of rflns unique	16995	26156	15841
no. of params / restraints	765 / 41	1149 / 18	780 / 22
R_1 ($I > 2\sigma(I)$) [a]	0.046	0.051	0.051
R_1 (all data)	0.057	0.078	0.074
wR_2 ($I > 2\sigma(I)$)	0.119	0.141	0.124
wR_2 (all data)	0.126	0.160	0.139
Diff.Fourier.peaks min/max, eÅ^{-3}	-1.94 / 2.45	-1.33 / 1.77	-4.04 / 1.52
CCDC number	2078523	2078526	2078525

5. Buried volume analysis

The steric description of per cent buried volume (%Vbur) has been shown to be a valid measure of the steric properties of monodentate ligands such as phosphines and NHCs. Comparison of related highly bulky ligands IPr**[5] and phosphine **1** is shown in Figure S28.[6]

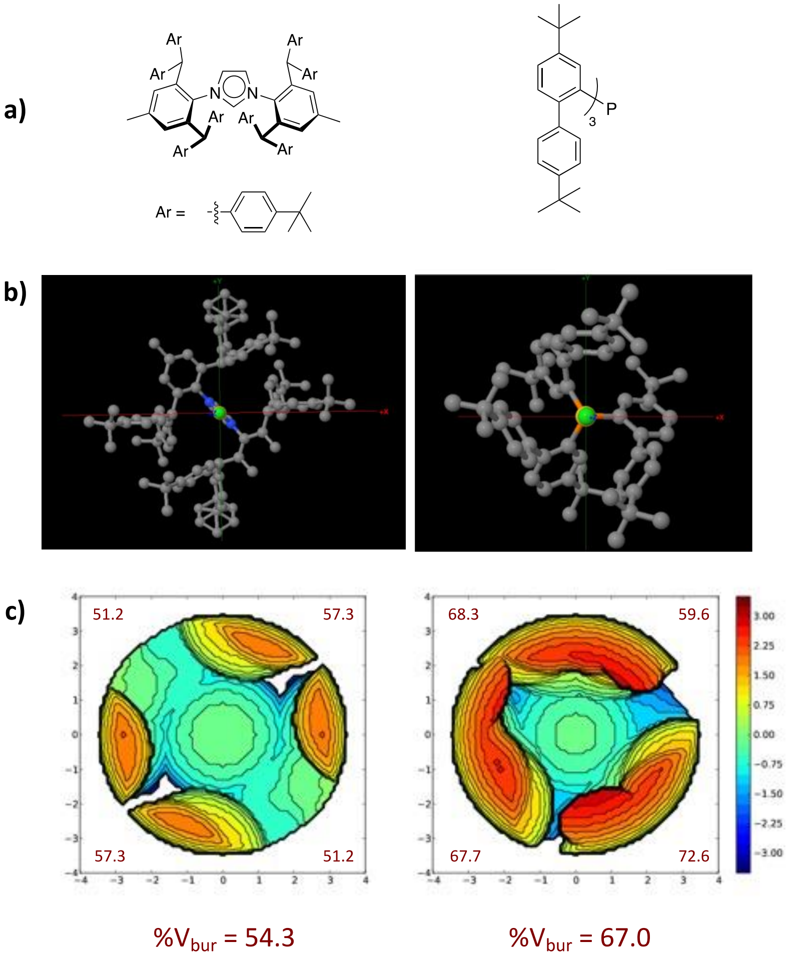

Figure S31. Schematic (a) and 3D representation (b) of the ligands, together with the corresponding steric maps (c) and calculated %Vbur for the IPr**AuCl and gold(I) complex **2**. The %Vbur of each quadrant are also indicated in red.

6. Computational details

Calculations were performed at the DFT level with the Gaussian 09 (Revision D.01) program.[7] The hybrid functional PBE0[8] was used throughout the computational study, and dispersion effects were accounted for by using Grimme's D3 parameter set with Becke−Johnson (BJ) damping at the optimisation stage.[9] Geometry optimisations were carried out without geometry constraints, using the 6-31G(d,p)[10] basis set to represent the C, H and P atoms and the Stuttgart/Dresden Effective Core Potential and its associated basis set (SDD)[11] to describe the Au atoms. Bulk solvent effects (dichloromethane) were included at the optimization stage with the SMD continuum model[12]. The stationary points and their nature as minima or saddle points (TS) were characterized by vibrational analysis, which also produced enthalpy (H), entropy (S) and Gibbs energy (G) data at 298.15 K. The minima connected by a given transition state were determined by perturbing the transition states along the TS coordinate and optimizing to the nearest minimum. Electronic energies were refined by means of single point calculations with the larger 6-311+G(2d,p) basis set and free energies were corrected (ΔG_{qh}) to account for errors associated with the harmonic oscillator approximation with the Goodvibes code.[13] Thus, according to Truhlars's quasi-harmonic approximation for vibrational entropy, all vibrational frequencies below 100 cm^{-1} were set to this value.[14] The NMR shieldings were calculated with the Gauge-Independent Atomic Orbital (GIAO)[15] method at the PBE0/6-311+G(2d,p)//PBE0/6-31G(d,p) level. The CYLview20 visualization software has been used to create some of the figures.[16]

6.1 Calculation of ^{1}H NMR chemical shifts

The average calculated ^{1}H NMR chemical shift relative to TMS for coordinated ethylene were 4.46 ppm for complex **4** and 6.45 ppm for $[PPh_3Au(C_2H_4)]^+$, supporting that the shift to higher field observed for the resonances of the ^{1}H nuclei of coordinated ethylene in **4** does not arise from Au→ethylene π-backdonation. The donor properties of PPh_3 and phosphine **1** are comparable, in agreement with the CO stretching frequency of the corresponding carbonyl complexes. In addition, attempts to prepare $[PPh_3Au(C_2H_4)]^+$ led to immediate decomposition and formation of $[(PPh_3)_2Au]^+$ and Au(0) under all screened conditions.

6.2 Ethylene exchange

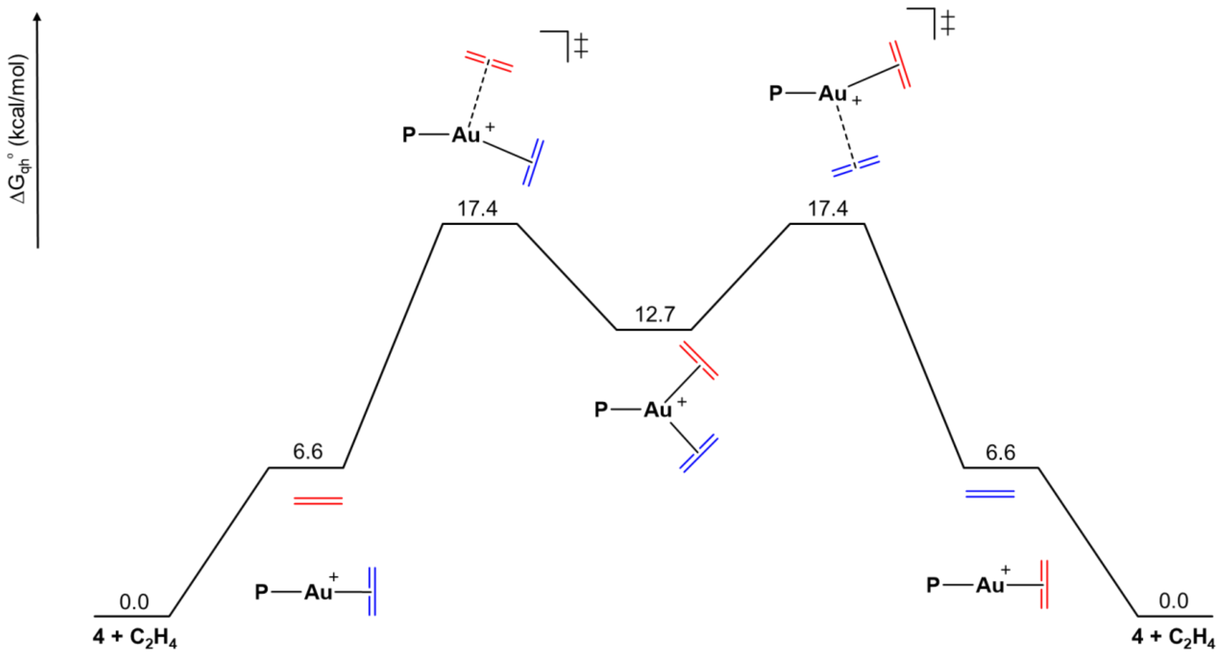

Figure S32. Free energy profile of the exchange between coordinated and free ethylene in complex **4**. Phosphine **1** has been represented as P for clarity.

6.3 Energy Decomposition Analysis Calculations

To enable a direct comparison with the available bonding analyses on related gold(I) complexes (see main text), the geometry of complex **4** (as well as complexes A and B) was re-optimized at the dispersion corrected BP86[17]-D3[18]/def2-SVP[19] level. The interaction between the transition metal fragment and ethylene has been investigated with the EDA-NOCV method,[20] which combines the energy decomposition analysis (EDA)[21] with the natural orbitals for chemical valence (NOCV)[22] methods. Within this approach, the interaction energy can be decomposed into the following physically meaningful terms:

$$\Delta E_{\text{int}} = \Delta E_{\text{elstat}} + \Delta E_{\text{Pauli}} + \Delta E_{\text{orb}} + \Delta E_{\text{disp}}$$

The term ΔE_{elstat} corresponds to the classical electrostatic interaction between the unperturbed charge distributions of the deformed reactants and is usually attractive. The Pauli repulsion ΔE_{Pauli} comprises the destabilizing interactions between occupied orbitals and is responsible for any steric repulsion. The orbital interaction ΔE_{orb} accounts for charge transfer (interaction between occupied orbitals on one moiety with unoccupied orbitals on the other, including HOMO–LUMO interactions) and polarization (empty-occupied

orbital mixing on one fragment due to the presence of another fragment). Finally, the ΔE_{disp} term takes into account the interactions which are due to dispersion forces.

The EDA-NOCV method makes it possible to further partition the total orbital interactions into pairwise contributions of the orbital interactions. Details of the method can be found in the literature.[23]

The EDA-NOCV calculations were carried out using the BP86-D3/def2-SVP optimized geometry with the program package ADF 2020[24] using the same functional (BP86-D3) in conjunction with a triple-ζ-quality basis set using uncontracted Slater-type orbitals (STOs) augmented by two sets of polarization function with a frozen-core approximation for the core electrons.[25] An auxiliary set of s, p, d, f, and g STOs were used to fit the molecular densities and to represent the Coulomb and exchange potentials accurately in each SCF cycle.[26] Scalar relativistic effects were incorporated by applying the zeroth-order regular approximation (ZORA).[27] This level of theory is denoted BP86-D3/TZ2P//BP86-D3/def2-SVP.

Table S3 EDA-NOCV data (in kcal/mol) computed for complexes **4**$^+$, $[(Ph_3P)Au(C_2H_4)]^+$ (**A**) and $[(NHC)Au(C_2H_4)]^+$ (**B**) and **6**$^+$.

	**4**$^+$	**A**	**B**	**6**$^+$
ΔE_{int}	−46.1	−38.6	−48.2	−41.4
ΔE_{Pauli}	142.4	106.4	106.7	183.8
ΔE_{elstat}[a]	−106.5 (56.5%)	−85.1 (58.7%)	−90.4 (58.4%)	−135.7 (60.2%)
ΔE_{orb}[a]	−66.9 (35.5%)	−57.2 (39.4%)	−61.6 (39.8%)	−78.3 (34.8%)
$\Delta E_{orb}(\rho_1)$[b]	−34.0 (50.8%)	−34.1 (59.6%)	−33.8 (58.9%)	−34.6 (44.2%)
$\Delta E_{orb}(\rho_2)$[b]	−18.4 (27.5%)	−14.3 (25.0%)	−18.1 (29.4%)	−30.0 (38.3%)
$\Delta E_{orb}(rest)$[b]	−14.5 (21.7%)	−8.8 (15.4%)	−9.7 (15.7%)	−13.7 (17.5%)
ΔE_{disp}[a]	−15.1 (8.0%)	−2.7 (1.9%)	−2.9 (1.9%)	−2.9 (1.9%)

[a] The values within parentheses indicate the percentage to the total attractive interactions, $\Delta E_{int} = \Delta E_{elstat} + \Delta E_{orb} + \Delta E_{disp}$.
[b] The values within parentheses indicate the percentage to the total orbital interactions (ΔE_{orb}).

6.4. Cartesian coordinates and energies for all the species discussed in the text

C2H4 E(RPBE1PBE) = -78.4888753201

C	0.000000000	0.664653000	0.000000000
H	0.925366000	1.236881000	0.000000000
H	-0.925317000	1.236936000	0.000000000
C	0.000000000	-0.664653000	0.000000000
H	-0.925366000	-1.236881000	0.000000000
H	0.925317000	-1.236936000	0.000000000

4+ E(RPBE1PBE) = -2884.55691516

Au	1.477650000	-0.011068000	-0.150729000
C	-3.349110000	2.928577000	1.303224000
C	-3.300053000	-2.591679000	2.007983000
C	-0.865586000	1.800496000	2.139217000
C	0.399689000	1.233929000	2.660159000
C	2.790863000	0.195089000	3.797836000
C	0.087057000	1.887899000	-2.443853000
C	-1.105467000	1.034758000	-2.654709000
C	-3.452079000	-0.503992000	-3.160997000
C	4.034277000	-0.387254000	4.469551000
C	2.375901000	3.528305000	-2.065770000
C	-4.689433000	3.529843000	0.888746000
C	-4.725341000	-1.323882000	-3.354536000
C	4.006689000	-3.731515000	-2.570645000
C	2.720473000	-3.449956000	-1.793720000
C	-0.913733000	-2.827295000	0.463322000
C	0.323604000	-3.028919000	-0.323220000
C	-5.672738000	3.411424000	2.062674000
H	-6.642146000	3.840364000	1.786030000
H	-5.830465000	2.362676000	2.337864000
H	-5.312093000	3.943152000	2.948878000
C	-5.291207000	2.815181000	-0.322691000
H	-4.640683000	2.878888000	-1.201921000

H	-5.484743000	1.757030000	-0.114547000
H	-6.246944000	3.281431000	-0.581891000
C	-4.489040000	5.010602000	0.535378000
H	-3.787335000	5.122730000	-0.298294000
H	-5.443720000	5.459342000	0.239439000
H	-4.100884000	5.581493000	1.384829000
C	-4.411690000	-2.811305000	-3.132619000
H	-4.033368000	-3.003381000	-2.123189000
H	-3.662756000	-3.166133000	-3.848729000
H	-5.320125000	-3.409088000	-3.266773000
C	-5.310262000	-1.150920000	-4.755706000
H	-6.220854000	-1.751530000	-4.847575000
H	-5.577582000	-0.108408000	-4.959530000
H	-4.612394000	-1.484974000	-5.530986000
C	-5.770255000	-0.866680000	-2.324707000
H	-5.416558000	-1.014125000	-1.298657000
H	-6.695065000	-1.441659000	-2.445404000
H	-6.009407000	0.194642000	-2.452702000
C	-2.769488000	1.860124000	0.623720000
H	-3.266456000	1.454813000	-0.249382000
C	-1.548142000	1.296887000	1.016957000
C	-1.434632000	2.897885000	2.799960000
C	-2.642028000	3.448033000	2.395998000
C	-2.793376000	-0.555431000	-1.929139000
H	-3.185675000	-1.202058000	-1.151652000
C	-2.893666000	0.310289000	-4.147646000
C	-1.745741000	1.057656000	-3.896269000
C	-1.644064000	0.188933000	-1.661130000
C	0.127106000	2.847862000	-1.422624000
C	1.250778000	3.641972000	-1.237162000
H	1.243463000	4.371840000	-0.432327000
C	2.314274000	2.595154000	-3.105896000
C	1.194103000	1.787022000	-3.291927000

C	1.511743000	2.053564000	2.877424000
H	1.460332000	3.105224000	2.607230000
C	2.682840000	1.541632000	3.433032000
H	3.516910000	2.217545000	3.588770000
C	1.675543000	-0.622783000	3.566302000
H	1.707125000	-1.672493000	3.845653000
C	0.503364000	-0.119207000	3.015086000
H	-0.354374000	-0.774324000	2.894519000
C	-2.659141000	-3.826373000	1.840801000
C	-2.708345000	-1.484094000	1.406143000
C	-1.496466000	-3.936050000	1.092765000
C	-1.537365000	-1.578310000	0.641690000
C	0.423872000	-2.644024000	-1.664208000
H	-0.431841000	-2.196362000	-2.161502000
C	1.601837000	-2.849129000	-2.380762000
H	1.631639000	-2.534236000	-3.418335000
C	2.603182000	-3.850610000	-0.454165000
C	1.433621000	-3.646839000	0.267402000
H	1.380365000	-3.956864000	1.307976000
C	4.101578000	4.162890000	-0.376642000
H	4.974901000	4.792012000	-0.171414000
H	4.398619000	3.117001000	-0.238131000
H	3.337429000	4.398680000	0.370602000
C	4.739132000	4.119027000	-2.780141000
H	5.589453000	4.768583000	-2.548601000
H	4.449395000	4.311238000	-3.818609000
H	5.085553000	3.082111000	-2.706819000
C	3.647343000	-0.867103000	5.877403000
H	3.255824000	-0.040333000	6.479722000
H	2.882635000	-1.649239000	5.839003000
H	4.524192000	-1.277588000	6.390399000
C	5.158265000	0.640788000	4.599478000
H	5.477672000	1.022883000	3.623567000

H	4.862979000	1.492569000	5.221296000
H	6.027307000	0.172226000	5.072653000
C	4.562177000	-1.581492000	3.660838000
H	3.798077000	-2.352600000	3.520334000
H	4.912813000	-1.264717000	2.673505000
H	5.407566000	-2.042408000	4.183757000
C	4.035013000	-3.003975000	-3.916212000
H	3.937691000	-1.919069000	-3.791173000
H	4.989531000	-3.197385000	-4.416419000
H	3.239401000	-3.344920000	-4.586191000
C	4.091525000	-5.244976000	-2.824070000
H	4.113229000	-5.804467000	-1.883155000
H	3.231310000	-5.594289000	-3.405092000
H	5.002460000	-5.486552000	-3.383335000
C	-4.321852000	-2.961261000	4.254190000
H	-5.240084000	-2.898222000	4.848290000
H	-3.978521000	-4.000259000	4.277298000
H	-3.559643000	-2.340676000	4.737518000
H	-0.921609000	3.306632000	3.666285000
H	-3.044029000	4.291478000	2.950196000
H	-3.074984000	-4.720667000	2.296510000
H	-1.027166000	-4.908248000	0.968969000
H	-3.159187000	-0.508628000	1.538152000
C	-4.588952000	-2.484284000	2.819198000
C	-5.659477000	-3.376795000	2.174374000
H	-5.867485000	-3.060649000	1.146169000
H	-5.349821000	-4.426293000	2.149435000
H	-6.592915000	-3.316459000	2.744708000
C	-5.119244000	-1.049988000	2.872014000
H	-4.406202000	-0.365614000	3.344969000
H	-5.352119000	-0.666219000	1.872507000
H	-6.042622000	-1.025482000	3.459335000
H	3.439107000	-4.337585000	0.039846000

C	3.596488000	4.409652000	-1.806903000
C	3.193436000	5.884262000	-1.954470000
H	2.401293000	6.159453000	-1.251150000
H	4.055295000	6.532448000	-1.760205000
H	2.832981000	6.091527000	-2.967737000
H	-1.344483000	1.699078000	-4.676186000
H	-3.353935000	0.380023000	-5.127595000
H	-0.735707000	2.984920000	-0.779382000
H	3.153827000	2.478221000	-3.782804000
H	1.182536000	1.059562000	-4.099749000
C	5.235588000	-3.290743000	-1.763011000
H	5.223240000	-2.212158000	-1.574782000
H	5.303527000	-3.803671000	-0.799226000
H	6.147760000	-3.523434000	-2.323235000
P	-0.847135000	-0.023420000	-0.029113000
C	3.658230000	-0.309462000	0.286762000
H	3.841307000	0.392251000	1.097417000
H	3.737857000	-1.369168000	0.517831000
C	3.553379000	0.121079000	-1.009829000
H	3.543049000	-0.586829000	-1.836103000
H	3.649016000	1.174174000	-1.262150000

4+·C2H4 E(RPBE1PBE) = -2963.05540240

Au	-1.461859000	-0.096826000	-0.151348000
C	3.393493000	-3.177262000	0.686006000
C	3.162463000	1.950439000	2.846066000
C	0.869954000	-2.346743000	1.724201000
C	-0.415867000	-1.949708000	2.341807000
C	-2.861127000	-1.236237000	3.605409000
C	0.148817000	-1.340611000	-2.734234000
C	1.294548000	-0.401321000	-2.666850000
C	3.520878000	1.380092000	-2.680990000
C	-4.146799000	-0.830172000	4.325710000
C	-2.053238000	-3.127922000	-2.908637000

C	4.753495000	-3.627528000	0.159474000
C	4.707433000	2.339766000	-2.623973000
C	-4.262056000	4.089241000	-1.140555000
C	-2.956965000	3.631752000	-0.491131000
C	0.727512000	2.493504000	1.459103000
C	-0.532713000	2.857962000	0.776999000
C	5.702536000	-3.844116000	1.347355000
H	6.684159000	-4.173354000	0.989090000
H	5.840041000	-2.915713000	1.912853000
H	5.324839000	-4.606753000	2.035644000
C	5.379953000	-2.594332000	-0.779108000
H	4.761799000	-2.416609000	-1.666145000
H	5.543711000	-1.634586000	-0.276469000
H	6.353572000	-2.958545000	-1.122170000
C	4.581270000	-4.945992000	-0.608838000
H	3.909608000	-4.814209000	-1.464230000
H	5.549948000	-5.291912000	-0.986258000
H	4.168407000	-5.734998000	0.027639000
C	4.228416000	3.705980000	-2.109891000
H	3.793284000	3.636234000	-1.107669000
H	3.473675000	4.134557000	-2.778242000
H	5.071833000	4.403532000	-2.059175000
C	5.352012000	2.534094000	-3.996339000
H	6.192217000	3.230290000	-3.909151000
H	5.740863000	1.593319000	-4.400457000
H	4.645737000	2.954297000	-4.720523000
C	5.762834000	1.774740000	-1.660725000
H	5.365234000	1.659992000	-0.646996000
H	6.623436000	2.450640000	-1.606321000
H	6.119723000	0.795653000	-1.998865000
C	2.808230000	-1.973811000	0.300506000
H	3.314829000	-1.342423000	-0.419802000
C	1.566152000	-1.553617000	0.794396000

C	1.444681000	-3.573114000	2.083700000
C	2.671394000	-3.980192000	1.579505000
C	2.806065000	1.109046000	-1.509675000
H	3.104542000	1.597108000	-0.588124000
C	3.090415000	0.750379000	-3.850088000
C	1.998104000	-0.113999000	-3.838445000
C	1.717524000	0.236365000	-1.480028000
C	0.136335000	-2.542502000	-2.014103000
C	-0.945755000	-3.408817000	-2.096477000
H	-0.916610000	-4.329164000	-1.519554000
C	-2.015794000	-1.946087000	-3.655726000
C	-0.938828000	-1.065517000	-3.569067000
C	-1.529043000	-2.793885000	2.286816000
H	-1.459202000	-3.735997000	1.748856000
C	-2.726778000	-2.441073000	2.906398000
H	-3.564302000	-3.127382000	2.838062000
C	-1.738689000	-0.398398000	3.659309000
H	-1.789323000	0.539004000	4.206425000
C	-0.540479000	-0.744183000	3.047353000
H	0.318159000	-0.086667000	3.142484000
C	2.423371000	3.114292000	3.098287000
C	2.641862000	1.061833000	1.909595000
C	1.240340000	3.373079000	2.423877000
C	1.446792000	1.308123000	1.217916000
C	-0.643116000	2.878666000	-0.616900000
H	0.215986000	2.623430000	-1.231915000
C	-1.833975000	3.258545000	-1.234660000
H	-1.864539000	3.265217000	-2.318220000
C	-2.838018000	3.607929000	0.907183000
C	-1.653814000	3.235093000	1.529594000
H	-1.595044000	3.227829000	2.614805000
C	-3.774492000	-4.291368000	-1.521331000
H	-4.614128000	-4.995250000	-1.529763000

H	-4.133673000	-3.342721000	-1.106640000
H	-3.013040000	-4.688928000	-0.843315000
C	-4.369267000	-3.598506000	-3.832929000
H	-5.193101000	-4.319327000	-3.813246000
H	-4.055621000	-3.483759000	-4.875842000
H	-4.762224000	-2.636621000	-3.484732000
C	-3.858918000	-0.745238000	5.832821000
H	-3.514643000	-1.709752000	6.221116000
H	-3.090999000	0.002729000	6.054765000
H	-4.768392000	-0.464056000	6.375597000
C	-5.276321000	-1.836348000	4.102983000
H	-5.525739000	-1.941514000	3.041128000
H	-5.023737000	-2.826902000	4.495778000
H	-6.177244000	-1.494813000	4.622749000
C	-4.618070000	0.543982000	3.826960000
H	-3.850653000	1.313103000	3.957944000
H	-4.886802000	0.509231000	2.766327000
H	-5.504971000	0.859520000	4.387552000
C	-4.206072000	4.002927000	-2.666181000
H	-4.022984000	2.978548000	-3.010251000
H	-5.163839000	4.327458000	-3.085626000
H	-3.425686000	4.648124000	-3.083425000
C	-4.524602000	5.550163000	-0.743238000
H	-4.623591000	5.661508000	0.341185000
H	-3.708479000	6.200133000	-1.076693000
H	-5.453149000	5.905843000	-1.203718000
C	4.198990000	1.639040000	5.093808000
H	5.131502000	1.456832000	5.639166000
H	3.777405000	2.580968000	5.458290000
H	3.497223000	0.834988000	5.340679000
H	0.919975000	-4.204376000	2.795734000
H	3.076865000	-4.936462000	1.898338000
H	2.777647000	3.836604000	3.828434000

H	0.697630000	4.292108000	2.627515000
H	3.166971000	0.134668000	1.715601000
C	4.472030000	1.684300000	3.583372000
C	5.457551000	2.820769000	3.274362000
H	5.668935000	2.876168000	2.200780000
H	5.067024000	3.792539000	3.592387000
H	6.404215000	2.651751000	3.799354000
C	5.109821000	0.357610000	3.165130000
H	4.456459000	-0.495681000	3.377860000
H	5.358367000	0.342488000	2.098044000
H	6.040526000	0.210007000	3.722215000
H	-3.683127000	3.894720000	1.527137000
C	-3.229943000	-4.101466000	-2.945765000
C	-2.747973000	-5.455441000	-3.486982000
H	-1.958730000	-5.882772000	-2.860489000
H	-3.579377000	-6.168736000	-3.513089000
H	-2.354584000	-5.353161000	-4.504009000
H	1.688739000	-0.596636000	-4.761546000
H	3.601610000	0.927449000	-4.790183000
H	0.986759000	-2.808605000	-1.396158000
H	-2.841587000	-1.686688000	-4.309789000
H	-0.947736000	-0.143512000	-4.144785000
C	-5.425567000	3.216031000	-0.649236000
H	-5.289182000	2.172577000	-0.951673000
H	-5.528086000	3.244148000	0.440037000
H	-6.367951000	3.571392000	-1.080774000
P	0.850271000	-0.018845000	0.110088000
C	-3.667358000	-0.034262000	0.256633000
H	-3.818887000	-0.893715000	0.905571000
H	-3.818982000	0.951128000	0.690944000
C	-3.510932000	-0.189963000	-1.095251000
H	-3.535625000	0.665521000	-1.766598000
H	-3.531765000	-1.175328000	-1.553006000

C	0.464414000	3.354432000	-4.002165000
H	0.177220000	4.321336000	-3.594871000
H	1.400824000	2.932316000	-3.645509000
C	-0.288276000	2.722542000	-4.900638000
H	0.008716000	1.761796000	-5.315761000
H	-1.223121000	3.142864000	-5.266004000

Exchange TS E(RPBE1PBE) = -2963.04505712

Au	1.177496000	-0.071397000	-1.063011000
C	-3.179453000	2.438688000	2.195850000
C	-3.171685000	-2.967697000	1.967375000
C	-0.470463000	1.539555000	2.303753000
C	0.949608000	1.164173000	2.471023000
C	3.658600000	0.551132000	3.071982000
C	-0.839759000	2.433916000	-1.839457000
C	-1.983227000	1.490103000	-1.999530000
C	-4.096749000	-0.326573000	-2.602974000
C	5.101370000	0.181825000	3.413626000
C	1.419132000	4.156823000	-1.625033000
C	-4.618063000	2.945007000	2.128263000
C	-5.197866000	-1.343794000	-2.893384000
C	4.749854000	-3.858001000	-1.453537000
C	3.343898000	-3.568693000	-0.928213000
C	-0.612457000	-2.948124000	0.706628000
C	0.747314000	-3.079500000	0.120161000
C	-5.299374000	2.716853000	3.484997000
H	-6.329726000	3.087890000	3.455043000
H	-5.329219000	1.650942000	3.735766000
H	-4.778624000	3.240768000	4.292698000
C	-5.431552000	2.232724000	1.046307000
H	-5.009160000	2.387922000	0.047351000
H	-5.496530000	1.154509000	1.229134000
H	-6.452059000	2.628634000	1.039336000
C	-4.598571000	4.447982000	1.812930000

H	-4.116179000	4.639252000	0.848055000
H	-5.622039000	4.836096000	1.764415000
H	-4.061293000	5.014752000	2.579814000
C	-4.553325000	-2.657068000	-3.361721000
H	-3.889057000	-3.076314000	-2.598836000
H	-3.968004000	-2.502270000	-4.274705000
H	-5.327699000	-3.401919000	-3.576463000
C	-6.158791000	-0.855633000	-3.977060000
H	-6.932600000	-1.611489000	-4.145097000
H	-6.659407000	0.074384000	-3.686865000
H	-5.646663000	-0.687612000	-4.930495000
C	-5.998967000	-1.602768000	-1.608289000
H	-5.368495000	-2.018561000	-0.815869000
H	-6.803458000	-2.320593000	-1.803547000
H	-6.452171000	-0.678392000	-1.233613000
C	-2.691207000	1.465106000	1.330541000
H	-3.355475000	1.074497000	0.570000000
C	-1.361166000	1.010769000	1.354053000
C	-0.964357000	2.529360000	3.170611000
C	-2.274728000	2.973596000	3.121383000
C	-3.152856000	-0.604757000	-1.605449000
H	-3.224914000	-1.543538000	-1.067178000
C	-3.968275000	0.882523000	-3.283493000
C	-2.926648000	1.761851000	-2.990023000
C	-2.122302000	0.274338000	-1.283122000
C	-0.643715000	3.232719000	-0.705514000
C	0.463504000	4.065737000	-0.603880000
H	0.578335000	4.661876000	0.297323000
C	1.184223000	3.408774000	-2.782043000
C	0.076486000	2.570144000	-2.889193000
C	1.914900000	2.171052000	2.579245000
H	1.620971000	3.207075000	2.435523000
C	3.242206000	1.869973000	2.868360000

H	3.953378000	2.685856000	2.941057000
C	2.688169000	-0.453030000	2.949223000
H	2.970276000	-1.490750000	3.099469000
C	1.362784000	-0.161165000	2.649317000
H	0.633459000	-0.964692000	2.593405000
C	-2.356641000	-4.107027000	1.962775000
C	-2.671437000	-1.832588000	1.338128000
C	-1.112351000	-4.088385000	1.355604000
C	-1.418471000	-1.795305000	0.701279000
C	0.952105000	-3.231350000	-1.251683000
H	0.100905000	-3.199210000	-1.923403000
C	2.227847000	-3.471822000	-1.763663000
H	2.335835000	-3.601013000	-2.835295000
C	3.124403000	-3.434911000	0.451241000
C	1.855116000	-3.207341000	0.966645000
H	1.711736000	-3.136017000	2.040691000
C	3.425043000	4.613650000	-0.208477000
H	4.302128000	5.255353000	-0.068078000
H	3.774295000	3.580250000	-0.312499000
H	2.816899000	4.676031000	0.699002000
C	3.575978000	5.003830000	-2.659122000
H	4.437333000	5.656085000	-2.482581000
H	3.082582000	5.347811000	-3.574350000
H	3.957331000	3.991563000	-2.833667000
C	5.130918000	-0.549899000	4.763775000
H	4.731584000	0.085573000	5.561479000
H	4.543554000	-1.473203000	4.742734000
H	6.161352000	-0.816301000	5.024629000
C	6.003956000	1.412121000	3.513426000
H	6.044707000	1.966085000	2.569186000
H	5.671725000	2.097751000	4.300321000
H	7.024160000	1.097757000	3.756475000
C	5.659622000	-0.741915000	2.321353000

H	5.054578000	-1.647402000	2.211588000
H	5.686154000	-0.232038000	1.352316000
H	6.681705000	-1.048824000	2.570603000
C	4.801496000	-3.880560000	-2.981511000
H	4.485833000	-2.924325000	-3.414332000
H	5.829079000	-4.068376000	-3.309152000
H	4.172009000	-4.671823000	-3.401981000
C	5.200233000	-5.230034000	-0.928725000
H	5.222130000	-5.253767000	0.165535000
H	4.525820000	-6.021994000	-1.271660000
H	6.208604000	-5.460468000	-1.290270000
C	-4.343457000	-3.329934000	4.138183000
H	-5.313642000	-3.353228000	4.646624000
H	-3.870578000	-4.307572000	4.273932000
H	-3.717779000	-2.579014000	4.632530000
H	-0.289474000	2.945161000	3.913338000
H	-2.594499000	3.744847000	3.816491000
H	-2.691787000	-5.021902000	2.443540000
H	-0.495306000	-4.982435000	1.371265000
H	-3.273537000	-0.933945000	1.345858000
C	-4.536080000	-2.990622000	2.653018000
C	-5.414366000	-4.062051000	1.991075000
H	-5.571642000	-3.841547000	0.929425000
H	-4.967033000	-5.057965000	2.068349000
H	-6.394892000	-4.097166000	2.478580000
C	-5.253977000	-1.643601000	2.553638000
H	-4.681165000	-0.841969000	3.032440000
H	-5.445746000	-1.360170000	1.512848000
H	-6.221576000	-1.708211000	3.061474000
H	3.957151000	-3.531420000	1.142660000
C	2.639796000	5.059138000	-1.451539000
C	2.174214000	6.510617000	-1.263837000
H	1.532501000	6.620207000	-0.384120000

H	3.039413000	7.170036000	-1.131991000
H	1.612486000	6.857323000	-2.137859000
H	-2.838128000	2.688804000	-3.550270000
H	-4.673248000	1.154241000	-4.061477000
H	-1.366329000	3.214398000	0.100862000
H	1.869714000	3.467074000	-3.620927000
H	-0.075252000	2.008627000	-3.806268000
C	5.726084000	-2.784097000	-0.952059000
H	5.435667000	-1.788233000	-1.303751000
H	5.777926000	-2.756880000	0.140162000
H	6.734636000	-2.993163000	-1.325582000
P	-0.902694000	-0.169161000	0.015707000
C	3.252599000	0.384597000	-0.362885000
H	3.155019000	1.345097000	0.135341000
H	3.514833000	-0.464746000	0.261147000
C	3.299475000	0.289113000	-1.735955000
H	3.624511000	-0.631368000	-2.212738000
H	3.254073000	1.180607000	-2.356487000
C	-0.100939000	-0.856716000	-3.608876000
H	-0.491114000	-1.833898000	-3.341965000
H	-0.834282000	-0.069300000	-3.747696000
C	1.207009000	-0.651290000	-3.818624000
H	1.583454000	0.315430000	-4.142170000
H	1.930147000	-1.457048000	-3.735681000

[P*Au(C2H4)2]+ E(RPBE1PBE) = -2963.05434183

Au	1.318843000	0.244027000	-1.051474000
C	-3.399426000	2.177827000	2.226899000
C	-2.955392000	-3.241341000	1.508114000
C	-0.723906000	1.258807000	2.593488000
C	0.632088000	0.781766000	2.938974000
C	3.200581000	-0.117020000	3.759418000
C	-0.799008000	2.533638000	-1.804437000
C	-1.857939000	1.515407000	-2.041775000

C	-3.876563000	-0.381079000	-2.710578000
C	4.583791000	-0.641749000	4.139188000
C	1.260096000	4.467063000	-1.438407000
C	-4.834891000	2.663137000	2.043163000
C	-4.963584000	-1.416016000	-2.992233000
C	4.928801000	-3.404937000	-2.089134000
C	3.498680000	-3.262433000	-1.571233000
C	-0.479339000	-2.974036000	0.106975000
C	0.868190000	-2.992641000	-0.514258000
C	-5.681093000	2.189806000	3.234113000
H	-6.713776000	2.539080000	3.124552000
H	-5.696571000	1.095945000	3.294479000
H	-5.296663000	2.575511000	4.183609000
C	-5.465003000	2.131008000	0.754620000
H	-4.914298000	2.455154000	-0.135424000
H	-5.515984000	1.036677000	0.746100000
H	-6.488349000	2.509688000	0.667110000
C	-4.839095000	4.197667000	1.988435000
H	-4.240297000	4.560298000	1.145679000
H	-5.862892000	4.566812000	1.862463000
H	-4.435415000	4.637475000	2.905757000
C	-4.329681000	-2.803905000	-3.167944000
H	-3.803199000	-3.134276000	-2.266780000
H	-3.615763000	-2.807260000	-3.998888000
H	-5.107282000	-3.544174000	-3.385471000
C	-5.761070000	-1.082732000	-4.253031000
H	-6.513022000	-1.859583000	-4.425040000
H	-6.289552000	-0.128107000	-4.160772000
H	-5.118771000	-1.037049000	-5.139227000
C	-5.925800000	-1.445457000	-1.794324000
H	-5.401333000	-1.703462000	-0.868555000
H	-6.712258000	-2.191558000	-1.955380000
H	-6.405060000	-0.470714000	-1.652141000

C	-2.774452000	1.343996000	1.304438000
H	-3.324958000	1.048828000	0.420807000
C	-1.451121000	0.892460000	1.445744000
C	-1.348030000	2.119811000	3.510780000
C	-2.643742000	2.576711000	3.335743000
C	-2.980424000	-0.601682000	-1.657004000
H	-3.065691000	-1.521054000	-1.089661000
C	-3.737729000	0.804992000	-3.428890000
C	-2.745641000	1.726018000	-3.098324000
C	-1.987684000	0.311072000	-1.306671000
C	-0.694376000	3.263902000	-0.613244000
C	0.315755000	4.200872000	-0.437199000
H	0.356693000	4.744270000	0.503060000
C	1.125491000	3.767759000	-2.641996000
C	0.115127000	2.825761000	-2.824188000
C	1.615990000	1.684850000	3.355463000
H	1.400956000	2.750399000	3.347144000
C	2.875081000	1.243727000	3.752421000
H	3.612073000	1.982459000	4.048816000
C	2.195685000	-1.017350000	3.379713000
H	2.393844000	-2.085661000	3.398927000
C	0.938423000	-0.582633000	2.976911000
H	0.176566000	-1.311473000	2.715347000
C	-2.249701000	-4.364933000	1.060770000
C	-2.409953000	-1.996041000	1.209139000
C	-1.045905000	-4.228030000	0.389179000
C	-1.207973000	-1.833167000	0.500307000
C	1.108507000	-3.728366000	-1.677696000
H	0.277798000	-4.212125000	-2.185732000
C	2.395676000	-3.849467000	-2.200466000
H	2.529406000	-4.424486000	-3.110602000
C	3.251519000	-2.537184000	-0.398003000
C	1.968111000	-2.397062000	0.117810000

H	1.823701000	-1.853762000	1.044428000
C	3.180990000	5.098626000	0.035297000
H	3.951977000	5.851633000	0.233541000
H	3.680878000	4.137291000	-0.124404000
H	2.560149000	5.014026000	0.932895000
C	3.303628000	5.632547000	-2.388469000
H	4.075690000	6.375494000	-2.163527000
H	2.780424000	5.962600000	-3.292177000
H	3.809675000	4.686288000	-2.610368000
C	4.459363000	-1.599861000	5.332608000
H	4.042276000	-1.085826000	6.205173000
H	3.815169000	-2.454307000	5.103038000
H	5.445980000	-1.989675000	5.606875000
C	5.545288000	0.484310000	4.518264000
H	5.696184000	1.187327000	3.691797000
H	5.189857000	1.046661000	5.388196000
H	6.521194000	0.059425000	4.774372000
C	5.172900000	-1.399163000	2.938272000
H	4.544688000	-2.245898000	2.644133000
H	5.280475000	-0.734848000	2.072980000
H	6.165568000	-1.790478000	3.188030000
C	4.991163000	-4.182365000	-3.404099000
H	4.417243000	-3.688387000	-4.195886000
H	6.030932000	-4.250065000	-3.740120000
H	4.612758000	-5.203877000	-3.292193000
C	5.764322000	-4.151535000	-1.038047000
H	5.791974000	-3.610968000	-0.086333000
H	5.354654000	-5.149493000	-0.848685000
H	6.795905000	-4.267544000	-1.388932000
C	-3.979979000	-4.177002000	3.577222000
H	-4.907980000	-4.312129000	4.143815000
H	-3.565692000	-5.168687000	3.370000000
H	-3.268421000	-3.637988000	4.211976000

H	-0.796390000	2.410097000	4.400616000
H	-3.073343000	3.238909000	4.082390000
H	-2.629372000	-5.363747000	1.257734000
H	-0.495508000	-5.117773000	0.096446000
H	-2.943743000	-1.106936000	1.519492000
C	-4.260864000	-3.399426000	2.283140000
C	-5.265642000	-4.182501000	1.425868000
H	-5.478869000	-3.656031000	0.489373000
H	-4.894398000	-5.181134000	1.175601000
H	-6.208958000	-4.303483000	1.969718000
C	-4.880149000	-2.049260000	2.649408000
H	-4.212394000	-1.450956000	3.278745000
H	-5.131621000	-1.462430000	1.758919000
H	-5.806124000	-2.213922000	3.209390000
H	4.076533000	-2.075068000	0.137591000
C	2.356113000	5.503605000	-1.195395000
C	1.703868000	6.870657000	-0.939522000
H	1.047883000	6.847284000	-0.063759000
H	2.474481000	7.628961000	-0.761227000
H	1.106690000	7.187743000	-1.801232000
H	-2.657983000	2.644425000	-3.672640000
H	-4.400874000	1.029524000	-4.257292000
H	-1.417729000	3.112564000	0.178768000
H	1.814883000	3.949568000	-3.459823000
H	0.042948000	2.299182000	-3.772623000
C	5.538299000	-2.014021000	-2.319709000
H	4.970530000	-1.452528000	-3.069892000
H	5.567720000	-1.423410000	-1.398303000
H	6.567758000	-2.111444000	-2.682101000
P	-0.791633000	-0.101642000	0.033873000
C	2.734497000	1.299820000	0.312149000
H	2.228656000	2.220454000	0.587726000
H	2.947946000	0.603313000	1.117409000

C	3.368075000	1.182409000	-0.911239000
H	4.083651000	0.385454000	-1.094487000
H	3.361674000	2.007274000	-1.619615000
C	0.590019000	-0.624861000	-3.085928000
H	0.295526000	-1.645675000	-2.864386000
H	-0.194205000	0.056134000	-3.401291000
C	1.914338000	-0.297125000	-3.235880000
H	2.210995000	0.652698000	-3.672304000
H	2.686279000	-1.052838000	-3.117670000

PPh3Au(C2H4)+ E(RPBE1PBE) = -1249.56860319

Au	1.862107000	0.003277000	0.012002000
C	-1.126197000	1.182906000	-1.198948000
C	-1.149910000	0.441053000	1.616635000
C	-0.502208000	-0.012843000	2.772106000
C	-2.855970000	1.471133000	2.976265000
C	-1.117677000	-1.630721000	-0.433487000
P	-0.452210000	0.000692000	0.000975000
C	-1.039097000	0.270671000	4.023200000
H	-0.536335000	-0.081787000	4.918932000
C	-2.214467000	1.013812000	4.125123000
H	-2.628740000	1.239991000	5.103333000
C	-2.327878000	1.186815000	1.719654000
H	-2.831372000	1.540881000	0.824970000
C	-2.272512000	0.893816000	-1.944842000
H	-2.769811000	-0.065677000	-1.837282000
C	-2.141168000	3.076184000	-2.970195000
H	-2.535791000	3.812726000	-3.664026000
C	-0.997387000	3.364644000	-2.226626000
H	-0.499417000	4.323132000	-2.339507000
C	-2.127740000	-4.107561000	-1.206841000
H	-2.521078000	-5.074731000	-1.505998000
C	-0.970418000	-3.612452000	-1.806297000
H	-0.460608000	-4.190900000	-2.571062000

C	-2.277461000	-2.127996000	0.168515000
H	-2.787170000	-1.552378000	0.935312000
C	4.030615000	-0.667579000	0.072382000
H	4.127161000	-1.287911000	-0.816114000
H	4.115854000	-1.165252000	1.036131000
C	4.025562000	0.698033000	-0.016909000
H	4.107100000	1.317959000	0.873621000
H	4.118887000	1.196261000	-0.979821000
H	-3.770633000	2.051109000	3.055884000
H	0.418277000	-0.586286000	2.688858000
C	-0.485490000	2.419369000	-1.344774000
H	0.410860000	2.636987000	-0.767992000
C	-2.775546000	1.843933000	-2.829974000
H	-3.665002000	1.618662000	-3.410812000
C	-0.461179000	-2.377977000	-1.419168000
H	0.445004000	-1.991745000	-1.880436000
C	-2.777680000	-3.367775000	-0.221443000
H	-3.677634000	-3.755051000	0.246971000

TMS E(RPBE1PBE) = -448.870842855

C	-0.712233000	-1.371672000	-1.078735000
H	-0.682015000	-2.338643000	-0.563128000
H	-1.756898000	-1.167274000	-1.340254000
H	-0.149087000	-1.475231000	-2.013551000
Si	0.000612000	0.000052000	0.000031000
C	-0.999607000	0.128475000	1.592205000
H	-0.608353000	0.918069000	2.244155000
H	-2.050427000	0.360150000	1.382700000
H	-0.972292000	-0.812038000	2.154615000
C	1.796177000	-0.395128000	0.414515000
H	2.405221000	-0.471731000	-0.493648000
H	2.238106000	0.383543000	1.046981000
H	1.878913000	-1.346843000	0.951993000
C	-0.084769000	1.638240000	-0.928053000

H	0.491034000	1.597521000	-1.859877000
H	-1.118775000	1.893819000	-1.187154000
H	0.318597000	2.458432000	-0.322855000

EDA-NOCV calculations (BP86-D3/def2-SVP)

4+ E = - -2884.487396

Au	-1.500820000	0.354197000	-0.102899000
C	1.365237000	-1.630386000	0.535853000
C	0.511062000	-2.588417000	1.137330000
C	0.977886000	-3.923615000	1.196174000
H	0.326213000	-4.687744000	1.648532000
C	2.244305000	-4.285352000	0.721439000
H	2.554922000	-5.339064000	0.796858000
C	3.124302000	-3.325700000	0.165050000
C	2.648875000	-2.007654000	0.078103000
H	3.278147000	-1.246577000	-0.399383000
C	-0.831961000	-2.261876000	1.683273000
C	-1.004719000	-1.281996000	2.686025000
H	-0.127539000	-0.733517000	3.066101000
C	-2.278465000	-1.010629000	3.216848000
H	-2.359306000	-0.250538000	4.006636000
C	-3.430180000	-1.694011000	2.764934000
C	-3.244229000	-2.663419000	1.749542000
H	-4.108843000	-3.229379000	1.369794000
C	-1.978702000	-2.947891000	1.221806000
H	-1.872014000	-3.693817000	0.419706000
C	4.521382000	-3.723076000	-0.344643000
C	5.288986000	-2.515068000	-0.926137000
H	5.453056000	-1.721842000	-0.166108000
H	4.762072000	-2.059853000	-1.791005000
H	6.287707000	-2.840838000	-1.280820000
C	5.340592000	-4.306379000	0.834784000
H	5.461376000	-3.555453000	1.643318000
H	6.352093000	-4.606410000	0.490110000

H	4.857665000	-5.203295000	1.273725000
C	4.364778000	-4.789887000	-1.458307000
H	3.770490000	-4.391225000	-2.306941000
H	3.859786000	-5.705730000	-1.088980000
H	5.359657000	-5.092040000	-1.846452000
C	-4.831493000	-1.472074000	3.370217000
C	-4.872541000	-0.247651000	4.308702000
H	-4.207440000	-0.374233000	5.187142000
H	-5.901600000	-0.102808000	4.695435000
H	-4.580445000	0.688231000	3.786108000
C	-5.880256000	-1.263932000	2.247816000
H	-5.658575000	-0.358722000	1.646107000
H	-6.890466000	-1.135640000	2.688481000
H	-5.933873000	-2.127711000	1.554809000
C	-5.203206000	-2.737184000	4.189058000
H	-5.234923000	-3.642163000	3.548267000
H	-6.202708000	-2.616566000	4.657134000
H	-4.463032000	-2.918122000	4.995383000
C	1.557885000	1.202719000	1.371314000
C	1.123760000	2.550022000	1.552479000
C	1.797183000	3.311101000	2.533463000
H	1.484889000	4.355653000	2.690535000
C	2.870393000	2.796531000	3.278524000
H	3.364290000	3.455381000	4.006343000
C	3.315250000	1.471869000	3.094056000
C	2.619869000	0.694111000	2.144958000
H	2.923360000	-0.352077000	1.999035000
C	0.013519000	3.188446000	0.790244000
C	-1.046810000	3.823073000	1.480147000
H	-1.056095000	3.812133000	2.581654000
C	-2.090841000	4.445716000	0.782983000
H	-2.894054000	4.926606000	1.363337000
C	-2.139440000	4.463701000	-0.633489000

C	-1.073063000	3.838826000	-1.314027000
H	-1.039787000	3.835664000	-2.412220000
C	-0.014931000	3.223956000	-0.622096000
H	0.814654000	2.787649000	-1.196869000
C	4.507379000	0.849294000	3.844495000
C	5.158482000	1.846876000	4.823670000
H	4.445280000	2.184724000	5.603786000
H	5.554047000	2.742142000	4.300574000
H	6.010915000	1.363569000	5.342851000
C	4.018890000	-0.386766000	4.643008000
H	4.866861000	-0.850968000	5.188450000
H	3.581629000	-1.164429000	3.983121000
H	3.247969000	-0.099239000	5.387557000
C	5.571576000	0.408536000	2.803813000
H	5.920706000	1.273223000	2.201744000
H	5.176167000	-0.356447000	2.102598000
H	6.452571000	-0.031897000	3.315563000
C	-3.314039000	5.149644000	-1.358648000
C	-3.382977000	6.636265000	-0.922628000
H	-4.215451000	7.152215000	-1.445027000
H	-2.440072000	7.165830000	-1.170166000
H	-3.556523000	6.744542000	0.167365000
C	-3.161517000	5.096644000	-2.892973000
H	-3.132431000	4.053482000	-3.272152000
H	-2.243487000	5.617377000	-3.235102000
H	-4.025305000	5.598269000	-3.374791000
C	-4.635433000	4.434339000	-0.975827000
H	-4.623740000	3.374530000	-1.306925000
H	-5.500165000	4.930428000	-1.463710000
H	-4.817984000	4.449163000	0.118437000
C	1.735135000	0.398647000	-1.438521000
C	1.283972000	-0.196081000	-2.643200000
C	2.068977000	0.004475000	-3.798371000

H	1.735989000	-0.440808000	-4.749138000
C	3.269149000	0.729157000	-3.747718000
H	3.856301000	0.838233000	-4.672796000
C	3.741125000	1.301369000	-2.542804000
C	2.937319000	1.135194000	-1.398715000
H	3.253351000	1.566136000	-0.438894000
C	0.052651000	-1.044735000	-2.690557000
C	-1.188598000	-0.513102000	-3.117096000
H	-1.240796000	0.539391000	-3.438897000
C	-2.340157000	-1.316853000	-3.144345000
H	-3.283476000	-0.869885000	-3.495158000
C	-2.312767000	-2.671892000	-2.737377000
C	-1.068941000	-3.188783000	-2.316846000
H	-0.981228000	-4.237389000	-2.000577000
C	0.092070000	-2.398271000	-2.298371000
H	1.043709000	-2.840210000	-1.966643000
C	5.094872000	2.033767000	-2.492744000
C	5.389938000	2.605784000	-1.089658000
H	4.625284000	3.345574000	-0.773500000
H	6.370546000	3.123296000	-1.094517000
H	5.441146000	1.809407000	-0.317593000
C	5.090600000	3.200484000	-3.512329000
H	4.932713000	2.846370000	-4.551361000
H	6.062941000	3.735010000	-3.488462000
H	4.290266000	3.931955000	-3.275963000
C	6.211796000	1.020357000	-2.855636000
H	6.224044000	0.171763000	-2.140033000
H	7.205720000	1.513528000	-2.823384000
H	6.077664000	0.602928000	-3.874357000
C	-3.595894000	-3.524885000	-2.799322000
C	-4.710751000	-2.851023000	-1.960566000
H	-5.637585000	-3.460895000	-1.985927000
H	-4.970807000	-1.843321000	-2.344102000

H	-4.396991000	-2.749454000	-0.900597000
C	-3.377371000	-4.950125000	-2.249305000
H	-2.619819000	-5.511200000	-2.833756000
H	-4.325228000	-5.523144000	-2.304150000
H	-3.058626000	-4.940031000	-1.185552000
C	-4.052045000	-3.631889000	-4.277569000
H	-3.267055000	-4.109169000	-4.899407000
H	-4.271853000	-2.636609000	-4.714998000
H	-4.974258000	-4.245149000	-4.355023000
C	-3.721625000	0.434297000	-0.519897000
H	-3.811380000	0.900601000	-1.515851000
H	-4.051559000	-0.613949000	-0.429814000
C	-3.465811000	1.201048000	0.605941000
H	-3.578859000	0.772305000	1.615920000
H	-3.324046000	2.292968000	0.537000000
P	0.813025000	0.076921000	0.121241000

A E = -1249.714923

Au	1.872230000	-0.032543000	0.008212000
C	4.070613000	-0.732862000	0.136282000
C	4.083990000	0.626690000	-0.110366000
H	4.193241000	1.020801000	-1.136369000
H	4.191513000	1.356908000	0.711421000
H	4.168852000	-1.464116000	-0.685716000
H	4.166958000	-1.129553000	1.162666000
P	-0.463317000	-0.004824000	0.000235000
C	-1.085428000	1.667997000	-0.391353000
C	-2.135338000	2.245965000	0.353672000
C	-0.525644000	2.356084000	-1.492431000
C	-2.623201000	3.513257000	-0.009997000
C	-1.023087000	3.618411000	-1.848839000
C	-2.070362000	4.197351000	-1.106909000
H	-2.569952000	1.711023000	1.212452000
H	0.293036000	1.900747000	-2.074573000

H	-3.441680000	3.967389000	0.569544000
H	-0.590146000	4.155332000	-2.706888000
H	-2.455550000	5.190579000	-1.385116000
C	-1.152114000	-1.149090000	-1.244605000
C	-2.245968000	-0.769531000	-2.051701000
C	-0.603774000	-2.447805000	-1.347898000
C	-2.787932000	-1.695313000	-2.959990000
C	-1.154905000	-3.365350000	-2.254856000
C	-2.245739000	-2.988654000	-3.061379000
H	-2.671091000	0.243309000	-1.973565000
H	0.250141000	-2.742432000	-0.714830000
H	-3.639652000	-1.401728000	-3.592770000
H	-0.729582000	-4.377453000	-2.336713000
H	-2.673188000	-3.708532000	-3.776480000
C	-1.149089000	-0.479436000	1.623999000
C	-0.566627000	0.062144000	2.792669000
C	-2.275577000	-1.325019000	1.713784000
C	-1.118313000	-0.237291000	4.047431000
C	-2.816957000	-1.622755000	2.976201000
C	-2.242179000	-1.080315000	4.139140000
H	0.314240000	0.721980000	2.720312000
H	-2.726801000	-1.749923000	0.803631000
H	-0.667191000	0.184767000	4.958641000
H	-3.694044000	-2.284065000	3.049579000
H	-2.669709000	-1.318685000	5.125383000

B E = -1058.903983

Au	0.000000000	0.000000000	1.256743000
C	0.000000000	0.000000000	-0.765308000
C	0.000000000	0.688575000	-2.919625000
C	0.000000000	-0.688575000	-2.919625000
C	-2.540586000	2.329506000	-1.110016000
C	-2.540586000	-2.329506000	-1.110016000
C	2.540586000	-2.329506000	-1.110016000

C	2.540586000	2.329506000	-1.110016000
C	0.000000000	2.451103000	-1.105695000
C	1.247698000	3.069910000	-0.862412000
C	-1.247698000	3.069910000	-0.862412000
C	1.220262000	4.387788000	-0.363692000
C	-1.220262000	4.387788000	-0.363692000
C	0.000000000	5.039110000	-0.120532000
C	0.000000000	-2.451103000	-1.105695000
C	1.247698000	-3.069910000	-0.862412000
C	-1.247698000	-3.069910000	-0.862412000
C	1.220262000	-4.387788000	-0.363692000
C	-1.220262000	-4.387788000	-0.363692000
C	0.000000000	-5.039110000	-0.120532000
N	0.000000000	1.087122000	-1.589276000
N	0.000000000	-1.087122000	-1.589276000
H	0.000000000	1.413865000	-3.739708000
H	0.000000000	-1.413865000	-3.739708000
H	2.171207000	-4.906668000	-0.165419000
H	-2.171207000	-4.906668000	-0.165419000
H	0.000000000	-6.070217000	0.265475000
H	2.171207000	4.906668000	-0.165419000
H	-2.171207000	4.906668000	-0.165419000
H	0.000000000	6.070217000	0.265475000
H	2.613264000	1.425088000	-0.467032000
H	2.619847000	1.979293000	-2.160867000
H	3.418076000	2.969110000	-0.898143000
H	2.619847000	-1.979293000	-2.160867000
H	2.613264000	-1.425088000	-0.467032000
H	3.418076000	-2.969110000	-0.898143000
H	-3.418076000	-2.969110000	-0.898143000
H	-2.613264000	-1.425088000	-0.467032000
H	-2.619847000	-1.979293000	-2.160867000
H	-2.619847000	1.979293000	-2.160867000

H	-2.613264000	1.425088000	-0.467032000
H	-3.418076000	2.969110000	-0.898143000
C	0.692776000	0.000000000	3.414226000
C	-0.692776000	0.000000000	3.414226000
H	-1.263207000	0.938593000	3.529915000
H	-1.263207000	-0.938593000	3.529915000
H	1.263207000	0.938593000	3.529915000
H	1.263207000	-0.938593000	3.529915000

6+ E = -2919.238458

Au	1.478249000	-0.014073000	-0.122927000
C	-3.271051000	-2.103309000	2.646746000
C	-3.475565000	-1.180719000	-2.993302000
C	-0.905171000	-2.647275000	1.093901000
C	0.287917000	-2.968293000	0.262075000
C	2.629896000	-3.336532000	-1.354489000
C	0.407375000	1.697317000	2.367158000
C	-0.838649000	2.247583000	1.764511000
C	-3.297397000	3.285623000	0.681208000
C	3.869505000	-3.461567000	-2.261043000
C	2.826432000	0.512576000	3.359502000
C	-4.570276000	-1.854706000	3.434641000
C	-4.624200000	3.775986000	0.073247000
C	3.661052000	3.779534000	-1.844778000
C	2.375621000	2.987520000	-2.155132000
C	-1.152500000	0.505965000	-2.764250000
C	0.036539000	1.398234000	-2.640388000
C	-5.647410000	-2.847136000	2.923781000
H	-6.603140000	-2.691784000	3.466506000
H	-5.837834000	-2.701554000	1.839801000
H	-5.342821000	-3.902735000	3.076049000
C	-5.097314000	-0.415791000	3.244004000
H	-4.367609000	0.344485000	3.593061000
H	-5.344863000	-0.196880000	2.184056000

H	-6.026656000	-0.277142000	3.832703000
C	-4.313229000	-2.089027000	4.944744000
H	-3.539018000	-1.393211000	5.329719000
H	-5.245201000	-1.919978000	5.523058000
H	-3.976789000	-3.124338000	5.156378000
C	-4.430903000	3.968113000	-1.453932000
H	-4.137303000	3.024328000	-1.958654000
H	-3.644827000	4.723515000	-1.662025000
H	-5.374853000	4.317870000	-1.921380000
C	-5.084747000	5.113436000	0.687567000
H	-6.044374000	5.427556000	0.229209000
H	-5.251065000	5.030549000	1.781735000
H	-4.351905000	5.926846000	0.506245000
C	-5.719357000	2.707588000	0.330256000
H	-5.469770000	1.734241000	-0.141365000
H	-6.689846000	3.041566000	-0.092102000
H	-5.858275000	2.533191000	1.417427000
C	-2.708022000	-1.129653000	1.801356000
H	-3.171714000	-0.136321000	1.737910000
C	-1.547436000	-1.381289000	1.036111000
C	-1.443086000	-3.606120000	1.980774000
C	-2.595991000	-3.344265000	2.732572000
C	-2.757907000	2.057876000	0.243281000
H	-3.278304000	1.491264000	-0.543832000
C	-2.558739000	4.000339000	1.648067000
C	-1.358260000	3.491353000	2.174612000
C	-1.553166000	1.543481000	0.755489000
C	0.379934000	0.480061000	3.088505000
C	1.564398000	-0.095442000	3.564947000
H	1.497536000	-1.047879000	4.112645000
C	2.839495000	1.749819000	2.676930000
C	1.655374000	2.333843000	2.188006000
C	1.541852000	-3.239789000	0.851098000

H	1.631546000	-3.258949000	1.948486000
C	2.688199000	-3.421103000	0.054681000
H	3.646674000	-3.606029000	0.559805000
C	1.356151000	-3.122944000	-1.934505000
H	1.251532000	-3.081448000	-3.029535000
C	0.208242000	-2.948289000	-1.150961000
H	-0.762687000	-2.775468000	-1.638866000
C	-2.903530000	-0.546529000	-4.121829000
C	-2.840484000	-0.973700000	-1.753990000
C	-1.771407000	0.273237000	-4.010955000
C	-1.698058000	-0.153681000	-1.631332000
C	-0.047395000	2.625677000	-1.949572000
H	-1.022338000	2.976406000	-1.581177000
C	1.101982000	3.394772000	-1.703675000
H	0.984347000	4.330694000	-1.140593000
C	2.440966000	1.786736000	-2.903711000
C	1.301441000	1.000450000	-3.137970000
H	1.393286000	0.046641000	-3.680920000
C	4.143517000	-1.644240000	3.419723000
H	5.075173000	-2.133940000	3.771046000
H	4.116638000	-1.714471000	2.313389000
H	3.293374000	-2.236631000	3.815105000
C	5.385120000	0.539172000	3.416058000
H	6.276361000	0.002237000	3.799673000
H	5.448484000	1.582427000	3.787932000
H	5.459900000	0.559259000	2.309283000
C	3.719108000	-4.724966000	-3.147575000
H	3.642943000	-5.638518000	-2.522876000
H	2.814383000	-4.675885000	-3.787481000
H	4.599020000	-4.836130000	-3.815190000
C	5.176426000	-3.582769000	-1.449659000
H	5.345755000	-2.702343000	-0.796184000
H	5.186690000	-4.492949000	-0.815407000

H	6.042073000	-3.654389000	-2.139048000
C	3.964242000	-2.204571000	-3.167117000
H	3.086069000	-2.106378000	-3.837295000
H	4.029290000	-1.274335000	-2.565102000
H	4.867217000	-2.258441000	-3.809975000
C	3.370971000	5.080219000	-1.067279000
H	2.893691000	4.878321000	-0.085158000
H	4.319756000	5.618435000	-0.867115000
H	2.713710000	5.767310000	-1.638822000
C	4.379413000	4.145142000	-3.168161000
H	4.675303000	3.245487000	-3.745176000
H	3.724965000	4.764005000	-3.815661000
H	5.302996000	4.723982000	-2.958401000
C	-4.421879000	-3.232746000	-4.101203000
H	-5.314764000	-3.881522000	-4.217830000
H	-4.136607000	-2.874279000	-5.111233000
H	-3.590757000	-3.858201000	-3.714052000
H	-0.952647000	-4.589713000	2.053500000
H	-2.984103000	-4.135387000	3.392590000
H	-3.353566000	-0.688404000	-5.116631000
H	-1.358518000	0.764054000	-4.906360000
H	-3.225226000	-1.473514000	-0.854942000
C	-4.732001000	-2.061059000	-3.134629000
C	-5.886801000	-1.199033000	-3.706427000
H	-6.121526000	-0.350985000	-3.029867000
H	-5.640521000	-0.780328000	-4.703485000
H	-6.804610000	-1.812693000	-3.820250000
C	-5.182977000	-2.647959000	-1.779665000
H	-4.406377000	-3.298852000	-1.326570000
H	-5.440515000	-1.853826000	-1.047417000
H	-6.089952000	-3.269100000	-1.924442000
H	3.408469000	1.432666000	-3.293311000
C	4.100602000	-0.168370000	3.896083000

C	4.062095000	-0.130727000	5.446254000
H	3.171370000	-0.657437000	5.845944000
H	4.964333000	-0.621019000	5.868206000
H	4.032192000	0.914955000	5.815417000
H	-0.815794000	4.061353000	2.945441000
H	-2.918552000	4.971712000	2.015571000
H	-0.581036000	-0.026582000	3.263291000
H	3.789214000	2.274609000	2.499796000
H	1.702956000	3.275892000	1.619111000
C	4.587173000	2.892665000	-0.974212000
H	4.086072000	2.619058000	-0.021335000
H	4.875049000	1.957933000	-1.496939000
H	5.523192000	3.433830000	-0.722720000
P	-0.887233000	-0.002024000	0.012545000
C	3.424624000	-0.198429000	-0.052933000
O	4.553381000	-0.364787000	0.046297000

7. References

1 J. Keller, C. Schlierf, C. Nolte, P. Mayer, B. F. Straub. *Synthesis* **2006**, *2*, 354.

2 A. Uson, M. Laguna, D. A. Briggs, H. H. Murray, J. P. Fackler. *Inorg. Synth.* **2007**, *26*, 85.

3 The concentration of CO and Ethylene were taken from International Union of Pure and Applied Chemistry: Solubility Data Series, Vol. 43 and IUPAC-NIST Solubility Database (F. Camacho Rubio, S. Delgado Diaz, R. Alvaro Alvarez. *Revista Ing. Quim.* **1980**, *12*, 83–87).

4 G. M. Sheldrick, *Acta Cryst*. **2008,** *A64*, 112.

5 S. G. Weber, F. Rominger, B. F. Straub. *Eur. J. Inorg. Chem.* **2012**, 2863.

6 L. Falivene, R. Credendino, A. Poater, A. Petta, L. Serra, R. Oliva, V. Scarano, L. Cavallo. *Organometallics* **2016**, *35*, 2286.

7 M. J Frisch, G. W. Trucks, H. B. Schlegel, G. E. Scuseria, M. A. Robb, J. R. Cheeseman, G. Scalmani, V. Barone, B. Mennucci, G. A. Petersson, H. Nakatsuji, M. Caricato, X. Li, H. P. Hratchian, A. F. Izmaylov, J. Bloino, G. Zheng, J. L. Sonnenberg, M. Hada, M. Ehara, K. Toyota, R. Fukuda, J. Hasegawa, M. Ishida, T. Nakajima, Y. Honda, O. Kitao, H. Nakai, T. Vreven, J. A. J. Montgomery, J. E. Peralta, F. Ogliaro, M. Bearpark, J. J. Heyd, E. Brothers, K. N. Kudin, V. N. Staroverov, R. Kobayashi, J. Normand, K. Raghavachari, A. Rendell, J. C. Burant, S. S. Iyengar, J. Tomasi, M. Cossi, N. Rega, J. M. Millam, M. Klene, J. E. Knox, J. B. Cross, V. Bakken, C. Adamo, J. Jaramillo, R. Gomperts, R. E. Stratmann, O. Yazyev, A. J. Austin, R. Cammi, C. Pomelli, J. W. Ochterski, R. L. Martin, K. Morokuma, V. G. Zakrzewski, G. A. Voth, P. Salvador, J. J. Dannenberg, S. Dapprich, A. D. Daniels, O. Farkas, J. B. Foresman, J. V. Ortiz, J. Cioslowski, D. J. Fox. Gaussian 09, Revision D.01, Gaussian, Inc.: Wallingford CT, **2013**.

8 J. P. Perdew, K. Burke, M. Ernzerhof. M. *Phys. Rev. Lett.* **1996**, *77*, 3865.

9 S. Grimme, J. Antony, S. Ehrlich, H. Krieg. *J. Chem. Phys*. **2010**, *132*, 154104.

10 a) W. J. Hehre, R. Ditchfield, J. A. Pople. *J. Phys. Chem.* **1972**, *56*, 2257; b) P. C. Hariharan, J. A. Pople. *Theor. Chim. Acta.* **1973**, *28*, 213; c) M. M. Francl, W. J. Pietro, W. J. Hehre, J. S. Binkley, M. S. Gordon, D. J. Defrees, J. A. Pople. J*J. Chem. Phys.* **1982**, *77*, 3654.

11 D. Andrae, U. Haeussermann, M. Dolg, H. Stoll, H. Preuss. *Theor. Chim. Acta* **1990**, *77*, 123.

12 A. V. Marenich, C. J. Cramer, D. G. Truhlar, *J. Phys. Chem. B* **2009**, *113*, 6378.

13 I. Funes-Ardoiz, R. S. Paton, GoodVibes v2.0.2. DOI: 10.5281/zenodo.595246.

14 R. F. Ribeiro, A. V. Marenich, C. J. Cramer, D. G Truhlar. *J. Phys. Chem. B* **2011**, *115*, 14556.

15 a) K. Wolinski, J. F. Hinton, P. Pulay. *J. Am. Chem. Soc.* **1990**, *112*, 8251; b) M. Häser, R. Ahlrichs, H. P. Baron, P. Weiss, H. Horn. *Theor. Chim. Acta* **1992**, *83*, 455.

16 CYLview20; Legault, C. Y., Université de Sherbrooke, 2020 (http://www.cylview.org).
17 (a) A. D. Becke, *Phys. Rev. A* **1988**, *38*, 3098. (b) J. P. Perdew, *Phys. Rev. B* **1986**, *33*, 8822.
18 S. Grimme, J. Antony, S. Ehrlich, H. Krieg, *J. Chem. Phys*. **2010**, *132*, 154104.
19 F. Weigend, R. Ahlrichs, *Phys. Chem. Chem. Phys*. **2005**, *7*, 3297.
20 M. P. Mitoraj, A. Michalak, T. Ziegler, *J. Chem. Theory Comput*. **2009**, *5*, 962.
21 For a review, see: M. von Hopffgarten, G. Frenking, *WIREs Comput. Mol. Sci*. **2012**, *2*, 43.
22 M. P. Mitoraj, A. Michalak, *J. Mol. Model*. **2007**, *13*, 347.
23 See, for instance: (a) M. P. Mitoraj, A. Michalak, T. Ziegler, *Organometallics* **2009**, *28*, 3727. (b) A. N. N. Thi, G. Frenking, *Chem. Eur. J.* **2012**, *18*, 12733. (c) M. Parafiniuk, M. P. Mitoraj, *Organometallics* **2013**, *32*, 4103.
24 ADF program: www.scm.com.
25 J. G. Snijders, E. J. Baerends,P. Vernoojs, P. *At. Data. Nucl. Data Tables* **1982**, *26*, 483.
26 A. Krijn, E. J. Baerends, E. J.; Fit Functions in the HFS-Method, Internal Report (in Dutch), Vrije Universiteit Amsterdam, The Netherlands, **1984**.
27 (a) E. van Lenthe, E. J. Baerends, J. G. Snijders, *J. Chem. Phys.* **1993**, *99*, 4597. (b) E. van Lenthe, E. J. Baerends, J. G. Snijders, *J. Chem. Phys.* **1994**, *101*, 9783. (c) E. van Lenthe, A. Ehlers, E. J. Baerends, *J. Chem. Phys.* **1999**, *110*, 8943.
